# The H4K20-mono-methyltransferase SETD8 promotes global accessibility of infecting herpes simplex virus genomes

**DOI:** 10.1128/jvi.01293-25

**Published:** 2025-11-19

**Authors:** Jesse H. Arbuckle, Andy A. Yanez, Syeda S. Baksh, Tovah E. Markowitz, Jodi L. Vogel, Alison A. McBride, Thomas M. Kristie

**Affiliations:** 1 Laboratory of Viral Diseases, National Institute of Allergy and Infectious Diseases, National Institutes of Health 35037, Bethesda, Maryland, USA; 2 Integrated Data Sciences Section, Research Technologies Branch, National Institute of Allergy and Infectious Diseases, National Institutes of Health 35037, Bethesda, Maryland, USA; Northwestern University Feinberg School of Medicine, Chicago, Illinois, USA

**Keywords:** SETD8, KMT5A, H4K20me1, chromatin accessibility, epigenetic regulation, herpesvirus, HSV-1, herpes simplex virus, HSV reactivation, RNAPII

## Abstract

Epigenetic modulation of herpes simplex virus (HSV) immediate early (IE) genes is a critical parameter governing lytic infection, latency, and viral reactivation. Multiple factors, including epigenetic complexes associated with the cellular transcriptional coactivator HCF-1, modulate the state of HSV-1 chromatin. Although some aspects of HSV-1 chromatin biology have been elucidated, many epigenetic factors controlling HSV-1 chromatin accessibility and transcriptional regulation remain unknown. To identify novel epigenetic regulators of the initial stage of HSV-1 infection, an epigenetic chemical probe library was screened for molecules that impacted viral IE gene expression. This screen identified several epigenetic inhibitors that modulated IE expression. Notably, UNC0379, an inhibitor of the histone H4K20 mono-methyltransferase SETD8, potently suppressed HSV-1 IE gene transcription during lytic infection. UNC0379 treatment also repressed HSV-1 reactivation in a mouse ganglia explant model, and topical application suppressed primary ocular infection *in vivo*. Importantly, SETD8 inhibition resulted in enhanced heterochromatin suppression and reduced viral genome accessibility, as evident by increased repressive H3K9me3 marks and decreased ATAC-seq signal across the HSV-1 genome. Reduced IE transcription was concomitant with disruption of H4K20me1 deposition and impaired recruitment of HCF-1 and RNA polymerase II (RNAPII). These findings are consistent with the established roles of SETD8 in promoting chromatin accessibility through H4K20me1 deposition and in regulating transcriptional elongation, suggesting that SETD8 may facilitate multiple steps in HSV-1 IE gene expression. Overall, these results identified SETD8 as an essential epigenetic modulator of HSV-1 chromatin accessibility and represent a promising novel antiviral therapeutic target.

**IMPORTANCE:**

Herpes simplex virus is a highly prevalent human pathogen, with persistence of the viral genome in sensory neurons serving as a reservoir for recurrent disease and viral shedding. Epigenetic pharmaceuticals have proven to be valuable tools in elucidating the chromatin landscape of the viral genome and its impact on viral gene expression. With the use of a chemical library screen, the histone methyltransferase SETD8 was identified as a key factor required for promoting accessibility of the herpesvirus genome to transcriptional regulators. Importantly, pharmacological inhibition of SETD8 suppressed herpes simplex virus lytic infection, reduced viral reactivation in sensory neurons, and when applied topically, inhibited primary ocular infection of mice. Collectively, these findings establish SETD8 as a critical regulator of viral gene expression during lytic infection and the initiation of reactivation from latency. These results highlight SETD8 as a potential novel target for antiviral therapy.

## INTRODUCTION

Herpes simplex virus (HSV) has a dual replication cycle. During the primary lytic phase, infection of epithelial cells at mucosal surfaces results in a lytic gene cascade and subsequent shedding of viral progeny to the termini of sensory neurons. Secondarily, HSV establishes a quiescent latent infection in sensory neurons, where the viral genome persists as a circular DNA episome for the life of the host. During latency, lytic gene transcription is minimal, while the viral gene product latency-associated transcript (LAT) is highly expressed (1, 2). LAT is a long noncoding RNA that contributes to the establishment of latency, suppressing neuronal apoptosis, and localizing genomes to the nuclear periphery to silence latent genomes (2–7). Latency is not static; various stress mediators can stimulate HSV to reenter the lytic cycle and shed viral particles at the initial site of infection.

Recurrent cycles of HSV latency-reactivation cause diseases of the oral and genital epithelia, as well as more severe outcomes such as herpes simplex encephalitis (HSE) and herpetic keratitis. The mortality rate of HSE in the absence of antiviral intervention is 70%, while herpetic keratitis is the primary source of viral-mediated blindness in the United States (8–10). HSV infections that occur *in utero* or during childbirth can result in HSE and developmental morbidities. Current antiviral therapies employ nucleoside analogs, such as acyclovir (ACV), to target the viral DNA polymerase. Antiviral treatment is administered during episodic and recurrent symptoms; however, drug resistance can arise in immunocompromised individuals (11–14).

Transcriptional initiation of HSV-1 immediate early (IE) genes is a key determinant for the progression of lytic infection. Upon entry into the nucleus, the HSV-1 genome is rapidly assembled into unstable nucleosome structures by the cellular epigenetic machinery containing histone chaperones and chromatin remodelers (15–20). Concurrently, transcription of IE genes is initiated by the viral transactivator VP16 in cooperation with an extended network of cellular transcription factors (19). The inherent dependence on IE gene transcription is demonstrated by studies analyzing IE deletion mutants (21, 22). These studies showed that native functions of the lytic cascade of IE genes, Early (E) genes, DNA replication, Late (L) gene expression, and viral assembly are attenuated when IE gene expression is disrupted.

Modulation of the chromatinized HSV-1 genome and components of the cellular epigenetic machinery control the fundamental activities of the lytic, latency, and reactivation cycles. The HSV-1 genome is initially assembled into heterochromatic structures containing repressive histone signatures (H3K9me3 and H3K27me2) (23–28). These modifications serve as dynamic binding platforms for chromatin readers to mediate sequential chromatin condensation. One key factor central to circumventing this initial repression is the cellular transcriptional coactivator Host Cell Factor-1 (HCF-1). HCF-1 serves as a scaffold that bridges multiple protein complexes to the promoters of IE genes (19, 20, 29). HCF-1 interacts with histone demethylases (JMJD2 and LSD1) that limit the accumulation of repressive H3K9-methylation and histone methyltransferases (SETD1A/MLL) that deposit active H3K4-methylation (24, 26, 30–32). Adding further complexity, HCF-1 also recruits components of the super elongation complex (SEC), thereby facilitating transcriptional elongation of IE genes, a critical step for HSV-1 lytic infection and latency reactivation (33, 34).

Chromatin accessibility and nucleosome positioning across the HSV-1 genome are key determinants of the HSV-1 replication program. It has also been established that HCF-1 is essential for IE gene expression during lytic infection as well as for *in vivo* reactivation from latency (35, 36). Mass spectrometry and yeast two-hybrid studies have defined HCF-1-containing complexes that are essential for IE gene transcription (34, 37). However, the full complement of components and mechanisms involved in the dynamic modulation of chromatin during IE gene transcriptional initiation remains largely unknown.

**Fig 1:**
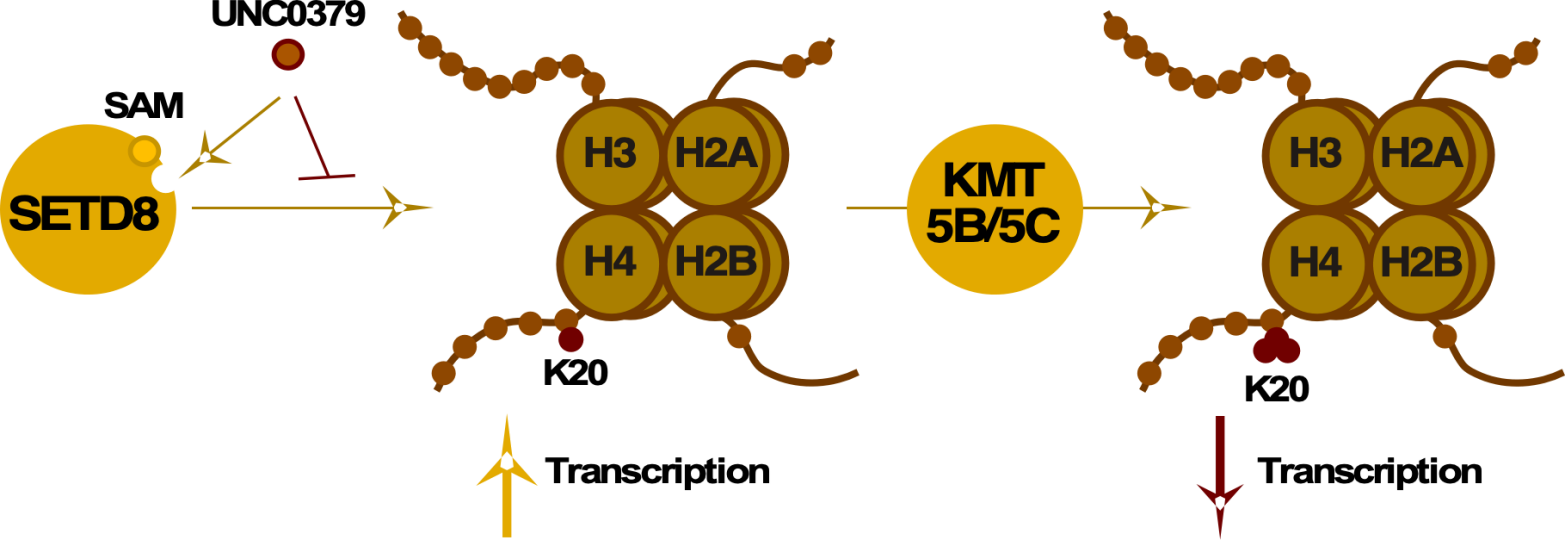
Schematic representation of the impacts of H4K20 methylation. SETD8-mediated H4K20 mono-methylation promotes gene transcription, while progression to H4K20 tri-methylation via KMT5B/C represses transcription (39). UNC0379 is a substrate competitive inhibitor of SETD8 (38).

To identify additional epigenetic factors that modulate the initial events of IE gene transcriptions, we screened an epigenetic chemical probe library in primary human fibroblast cells infected with HSV-1 for 1.5 hours (h). This screen identified several epigenetic modulators. In particular, the chemical probe UNC0379 (38), which targets the methyltransferase SETD8 (KMT5A, SET8, and PR-Set7), potently suppressed IE gene transcription. SETD8-mediated mono-methylation of H4K20 is known to stimulate cellular gene expression by promoting chromatin accessibility and transcription elongation (Fig. 1) (39–41). Using UNC0379, we investigated the role of SETD8 in HSV-1 IE transcription and found that UNC0379 suppressed IE expression during lytic infection and blocked the initiation of latency reactivation in sensory ganglia. Suppression of IE expression was concomitant with reduced HSV-1 genome accessibility and diminished recruitment of HCF-1 and RNAPII. We propose a model in which SETD8-mediated H4K20-monomethylation is essential for promoting HSV-1 genome accessibility and limiting heterochromatin accumulation. Most significantly, *in vivo* topical application of UNC0379 suppressed HSV-1 yields in an ocular mouse model, identifying SETD8 as a potential novel therapeutic target.

## RESULTS

### Epigenetic inhibitor screen revealed requirement for SETD8 in HSV-1 IE gene transcription and productive infection

Chromatin character and accessibility of the HSV-1 genome influences the progression of lytic infection (15, 16, 19, 20, 23–26, 29–32, 35, 36). Similarly, modulation of viral chromatin in sensory neurons plays regulatory roles in latency and viral reactivation. Although some aspects of HSV-1 chromatin biology have been elucidated, many of the characteristics and relevant chromatin modulation machinery remain unknown. Therefore, we screened a curated epigenetic inhibitor library for impacts on HSV-1 IE gene transcription. This library, obtained from the National Center for Advancing Translational Sciences (NCATS) and supplemented with additional compounds from commercial sources, comprised 48 compounds targeting a select group of chromatin readers, chromatin remodelers, methyltransferases, and demethylases, as listed in Table S1. In this screen, HFF fibroblast cells were pretreated for 4 h with epigenetic inhibitors or vehicle control and infected with HSV-1 for 1.5 h. Inhibitors were titrated across 3 to 7 dilutions, ranging from 0.01 µM to 25 µM, as defined by the literature. HSV-1 IE gene ICP27 transcript levels were determined by RT-QPCR and normalized to cellular controls (SP1 or GAPDH).

**Fig 2:**
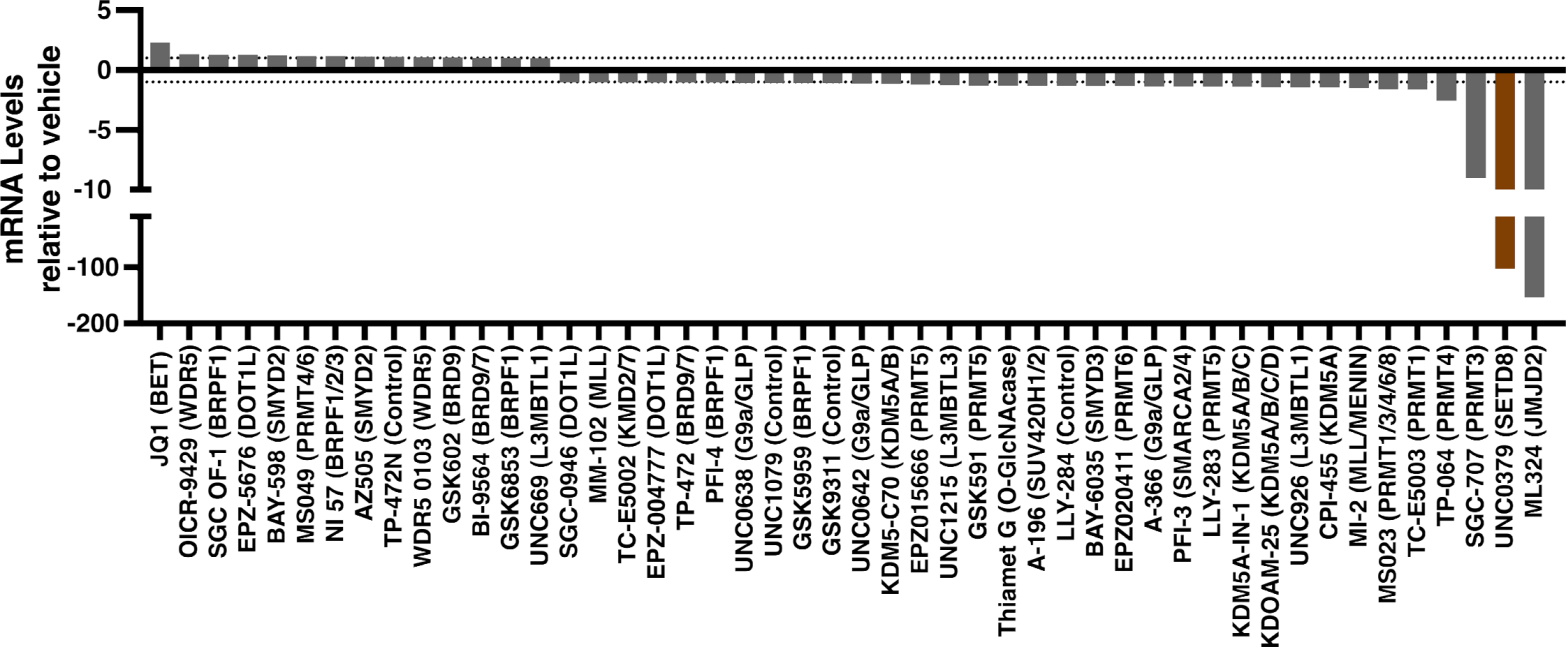
Epigenetic inhibitor screen identified compounds that suppressed HSV-1 IE gene expression. HFF cells were pretreated with the indicated inhibitors and infected with HSV-1 (MOI 1) for 1.5 h. mRNA levels of the viral IE ICP27 are relative to those in vehicle-treated cells and normalized to the cellular control genes SP1 or GAPDH. Results represent the highest inhibitor concentration utilized in the titration, as shown in Table S2. The intended target of each inhibitor is indicated in parentheses. The dotted line marks relative expression values 1 and −1. Data are from ≥ 2 replicate samples.

**Fig 3:**
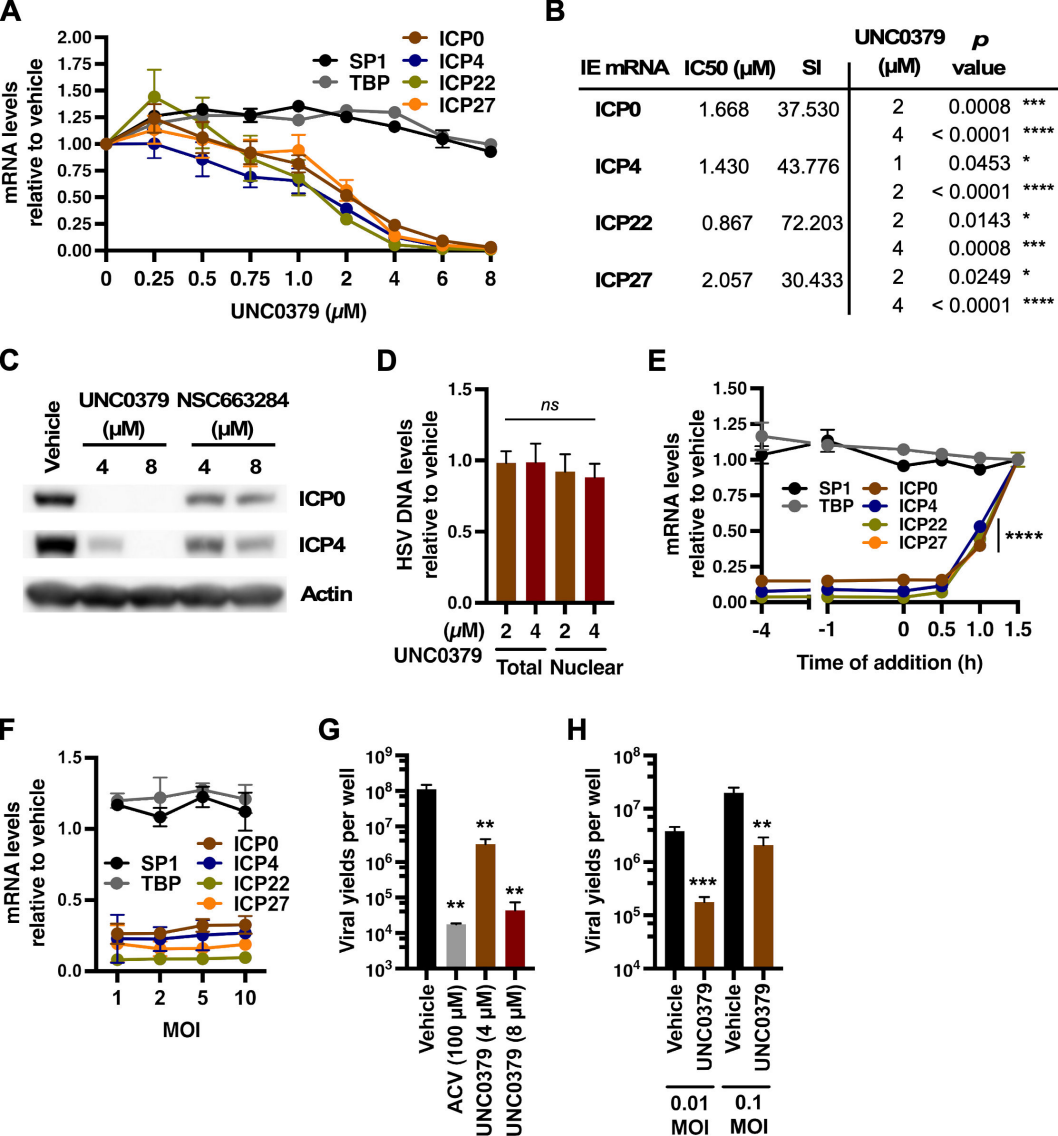
Inhibition of SETD8 suppressed transcription of HSV-1 IE genes. (**A**) HFF cells were treated with the indicated concentrations of UNC0379 and infected with HSV-1 [multiplicity of infection (MOI) 1]. mRNA levels of viral IE genes (ICP0, ICP4, ICP22, and ICP27) and cellular control genes (SP1 and TBP) are shown relative to levels in vehicle treated cells at 1.5 hpi. Data are from three experiments (4–6 replicate samples). (**B**) IC50 and SI values for UNC0379 inhibition of viral IE gene expression and *P*-values at various inhibitory concentrations of UNC0379. (**C**) Representative Western blot of viral IE and cellular (actin) proteins from HFF cells infected with HSV-1 (MOI 2) for 3 h and treated with vehicle, UNC0379, or NSC663284. (**D**) HFF cells were treated with vehicle or the indicated concentration of UNC0379 and infected with HSV-1 (MOI 1) for 1.5 h. Total and nuclear viral DNA levels in UNC0379 treated cells are shown relative to vehicle. Data from three experiments (9 replicate samples). (**E**) mRNA levels of viral IE and cellular control genes in HFF cells treated with 4 µM UNC0379 for the indicated duration relative to the time of infection with HSV-1 (MOI 1) for 1.5 h. RNA levels are shown relative to vehicle-treated cells. Data from three experiments (≥ 8 replicate samples). (**F**) HFF cells were treated with vehicle or 4 µM UNC0379 and HSV-1 infected at the indicated MOI for 1.5 h. The levels of viral IE and cellular control mRNAs in UNC0379-treated cells are shown relative to vehicle. Data are from three experiments (8 replicate samples). (**G**) Viral yields from HFF cells treated with vehicle, ACV, or UNC0379 and infected with HSV-1 (MOI 1) for 12 h. Data are from four experiments (12 replicate samples) and displayed as PFU/well. (**H**) Viral yields from HFF cells infected with HSV-1 at the indicated MOI for 8 h followed by an additional 12 h in the presence of vehicle or 4 µM UNC0379. Data are from three experiments (≥ 7 replicate samples) and displayed as PFU/well. *, *P* < 0.05; **, *P* < 0.01; ***, *P* < 0.001; ****, *P* < 0.0001; ns, not significant.

Several epigenetic modulators of HSV-1 IE transcription were identified, including SGC-707 (targeting PRMT3), TP-064 (targeting PRMT4), TC-E5003 (targeting PRMT1), MS023 (targeting PRMT1, -3, -4, -6, and -8) and UNC0379, a chemical probe targeting the H4K20 mono-methyltransferase SETD8 (Fig. 2; Table S2) (38, 42–45). UNC0379 was prioritized for further investigation due to its profound impact on ICP27 transcription, which was comparable to that of the previously established JMJD2 inhibitor ML324 and shown to suppress HSV-1 IE gene expression and latency reactivation (26). UNC0379 potently suppressed HSV-1 IE gene transcription and protein levels in a dose-dependent manner in both HFF and MRC-5 cells (Fig. 3A through C; Fig. S1 and S2). There was no apparent cytotoxicity at the evaluated concentrations or significant impact on cellular control genes (Fig. 3A through C; Fig. S3). Importantly, this suppression was not due to inhibition of viral entry as HSV-1 DNA levels in nuclear and total cell fractions were equivalent between UNC0379 and vehicle treatment (Fig. 3D). Rather than pretreating cells at 4 h prior to infection, treatment of cells at 1 hour post-infection (hpi) sufficiently reduced IE transcript levels (Fig. 3E). We also established that the suppression of HSV-1 was not MOI-dependent as increased viral input resulted in similar levels of inhibition (Fig. 3F). Additionally, treatment with NSC663284, a second compound targeting SETD8 (46), also suppressed IE gene transcription in a dose-dependent manner (Fig. 3C; Fig. S2 and S4).

**Fig 4:**
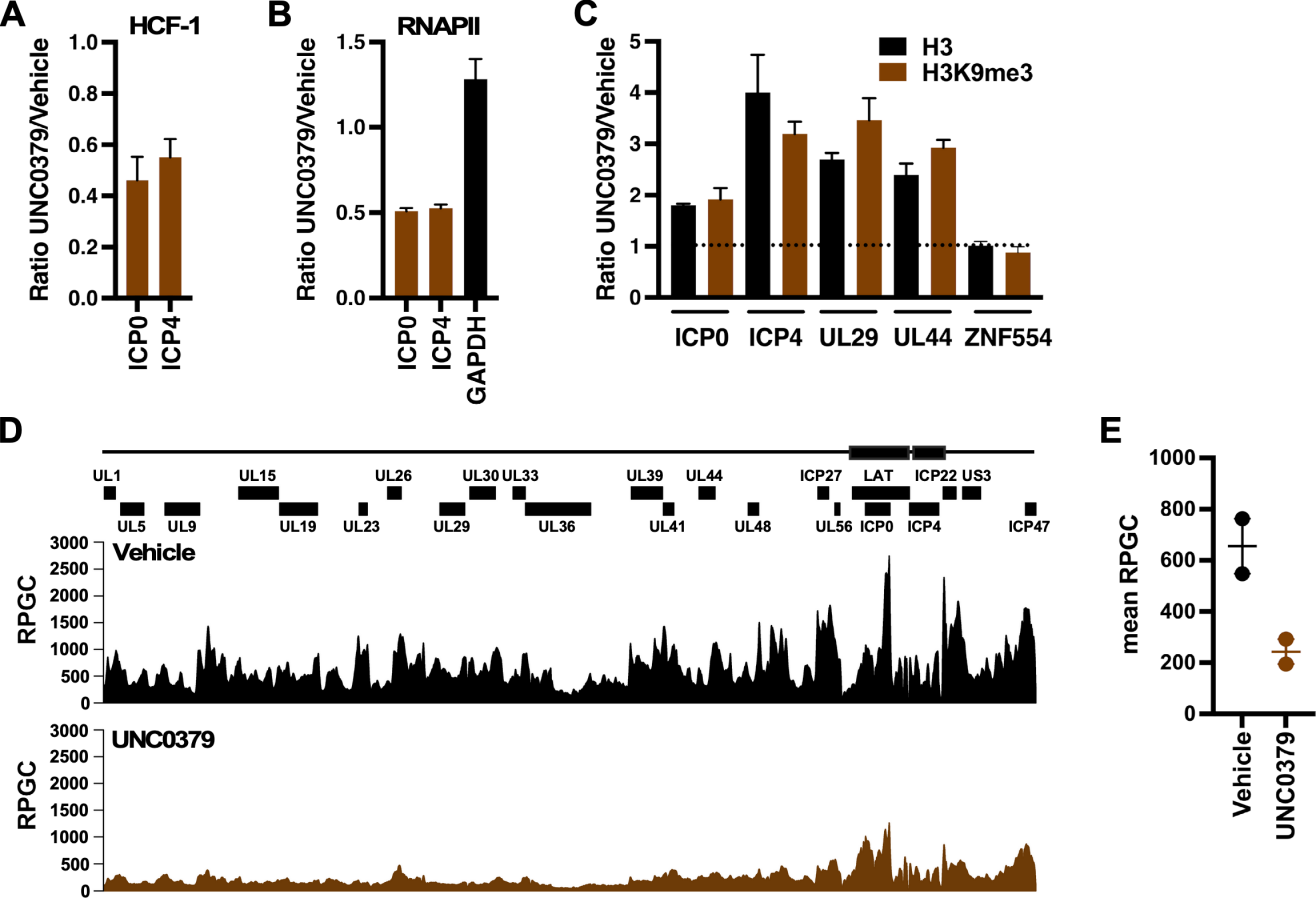
UNC0379 increased the levels of heterochromatin and reduced RNAPII occupancy across the HSV-1 genome. Levels of HCF-1 (**A**), RNAPII (**B**), and histones (H3, H3K9me3) (**C**) associated with HSV-1 gene promoters (ICP0, ICP4, UL29, and UL44) and cellular gene controls (GAPDH and ZNF554), as determined by ChIP-QPCR assays 2 hpi (MOI 5). Data are from three experiments and shown as the ratio of levels in 4 µM UNC0379 treated HFF cells relative to the vehicle. (**D**) Levels of RNAPII across the HSV-1 genome in vehicle and 4 µM UNC0379-treated HFF cells as determined by ChIP-seq. Data are from two independent experiments. (**E**) RNAPII ChIP-seq Mean reads per genomic cell (RPGC) for HSV-1 scaled genome-wide in vehicle- or UNC0379-treated samples.

To determine if SETD8 inhibition would suppress viral yields, HFF cells were pretreated with vehicle, SETD8 inhibitor UNC0379, or acyclovir (ACV, viral DNA polymerase inhibitor) for 12 h (Fig. 3G). Here, 8 µM UNC0379 treatment resulted in a 2.6 log reduction in viral yields as compared to vehicle and was nearly as effective as 100 µM ACV in plaque assays. Our results also indicated SETD8 inhibition reduced viral spread when cells were HSV-1-infected for 8 h to allow one replication cycle to occur before UNC0379 treatment for an additional 12 h (Fig. 3H). It was also apparent that suppression was not restricted to the alpha herpesvirus subfamily. UNC0379 treatment suppressed the transcription of UL123 (IE) and representative E and L gene expressions for the beta herpesvirus HCMV (Fig. S5) (47).

### UNC0379 suppressed HCF-1 coactivator complex recruitment to viral IE promoter/enhancer regions

The transition from heterochromatin to accessible euchromatin is essential for IE gene transcription. One particularly important component in this transition is the cellular transcriptional coactivator HCF-1 and its dynamic modulation of the chromatinized HSV-1 genome. This, in part, is mediated through HCF-1 recruitment of histone demethylases JMJD2 and LSD1 that limit the accumulation of repressive H3K9 methylation at IE promoters (24, 26, 32). Given that SETD8 inhibition reduced IE gene expression and viral yields, we employed chromatin immunoprecipitation (ChIP) to assess the impact on the HSV-1 chromatin. We found that UNC0379 treatment reduced HCF-1 recruitment to the ICP0 and ICP4 IE promoter/enhancer regions (Fig. 4A). Consistent with reduced viral IE transcription and HCF-1 recruitment, there was a parallel decrease in RNAPII recruitment (Fig. 4B). Importantly, total histone H3 levels and the heterochromatin mark H3K9me3 were increased in all classes of representative viral genes (IE = ICP0, ICP4; E = UL29; L = UL44) in cells treated with UNC0379 (Fig. 4C).

It is important to emphasize that IE expression is essential for progression of the lytic cascade (2, 19–22). The inhibition of SETD8 promoted heterochromatin formation across all classes of representative viral genes (Fig. 4C), while RNAPII recruitment was decreased at IE promoters, as determined by ChIP-QPCR (Fig. 4B). To further characterize RNAPII distribution across the HSV-1 genome, ChIP-seq was completed on HFF cells treated with vehicle or UNC0379 and infected for 2 h. Consistent with a previous study (48), RNAPII was distributed across the viral genome with elevated peaks found at IE genes (ICP27, ICP0, ICP4, ICP22, and ICP47) with vehicle treatment (Fig. 4D). In UNC0379-treated cells, global RNAPII recruitment was reduced. Mean reads per genomic content (RPGC) clearly demonstrated a 2.7-fold decrease in RNAPII recruitment across the HSV-1 genome with UNC0379 (Fig. 4E). These results highlight a critical role for SETD8 in HSV-1 IE transcription, which in part was mediated through recruitment of HCF-1 and RNAPII as well as reduced heterochromatin (H3K9me3) accumulation.

### SETD8 promoted chromatin accessibility of the HSV-1 genome

**Fig 5:**
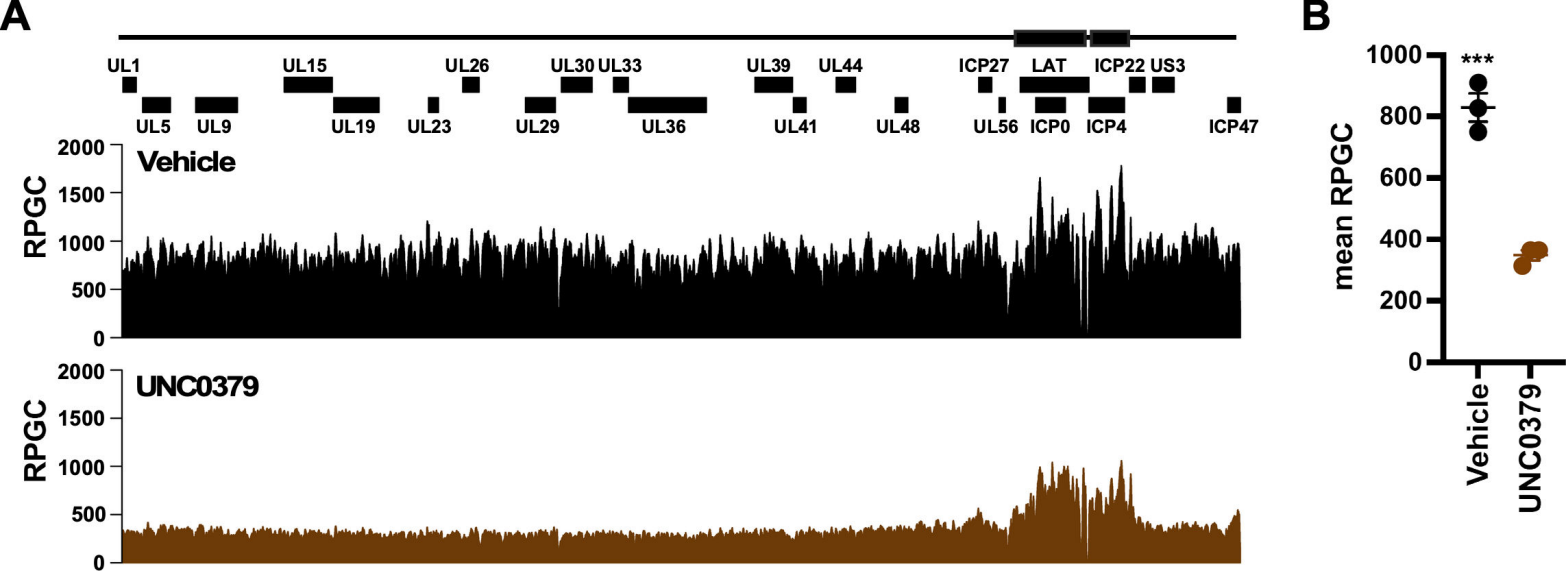
UNC0379 decreased global accessibility across the HSV-1 genome. Levels of accessible chromatin across the HSV-1 genome as measured by ATAC-seq at 1.5 hpi in vehicle- and 4 µM UNC0379-treated HFF cells. Data from three independent experiments. (**A**) The distribution of ATAC-seq RPGC traces across the HSV-1 genome. (**B**) Mean RPGC scaled values across the HSV-1 genome in vehicle- or UNC0379-treated samples. ***, *P* < 0.001.

One mechanism by which SETD8 promotes cellular gene expression is by facilitating chromatin accessibility through H4K20-monomethylation. Remarkably, it has been shown that H4K20me1 increases H4 tail flexibility, resulting in greater inter-nucleosomal distance and more mobile histone H4 (39). We hypothesized that SETD8 promotes HSV-1 IE transcription by increasing chromatin accessibility to the HCF-1 coactivator complex and other transcriptional regulators. To test this, we performed ATAC-seq in vehicle- or UNC0379-treated HFF cells infected with HSV-1 for 1.5 h (Fig. 5A). As anticipated, in vehicle-treated cells, the largest ATAC-seq peaks (and therefore accessible chromatin) were enriched in regions of the HSV-1 genome that overlapped with RNAPII ChIP-seq peaks (Fig. 4D), such as within the IE genes ICP0 and ICP4. Concomitant with increased H3 density and repressive H3K9me3 (Fig. 4C), UNC0379 reduced ATAC-seq reads across the HSV-1 genome (Fig. 5A). Most significantly, UNC0379 treatment decreased global chromatin accessibility across the HSV-1 genome by 2.6-fold compared to the vehicle, as measured by mean RPGC (Fig. 5B), without affecting viral genome levels. These results demonstrate that SETD8 promoted HSV-1 chromatin accessibility during lytic infection.

**Fig 6:**
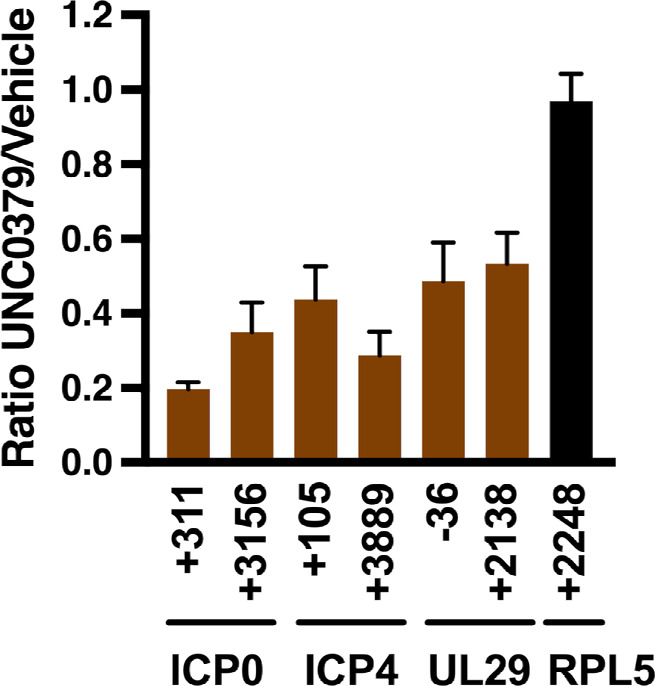
UNC0379 reduced the levels of histone H4K20me1 associated with the HSV-1 genome upon infection. Levels of H4K20me1 associated with HSV-1 (ICP0, ICP4, and UL29) and control cellular genes (RPL5), as determined by ChIP-QPCR assays 1 hpi. Data are shown as the ratio of levels in 8 µM UNC0379-treated HFF cells relative to the vehicle. The X-axis displays the positioning relative to the TSS for the indicated genes. Data are from three experiments.

We next explored whether reduced chromatin accessibility was initiated by a change in H4K20me1 levels on the HSV-1 genome. To this end, HFF cells were pretreated with vehicle or UNC0379 for 4 h, and chromatin was prepared at 1 hpi to capture the early stages of lytic infection. ChIP-QPCR assays revealed an UNC0379-mediated decrease in H4K20me1 levels proximal to the transcriptional start site (TSS) and regions further downstream within representative IE (ICP0 and ICP4) and E (UL29) viral genes (Fig. 6). It is important to note that decreased H4K20me1 levels were restricted to HSV-1 as no impact was observed on the cellular control gene RPL5. However, as determined through Western blot analysis, prolonged UNC0379 treatment (24 and 48 h) also reduced H4K20me1 levels in acid-extracted histones (Fig. S6), consistent with previous studies (49, 50). Collectively, ChIP results suggest that SETD8 directly modulated HSV-1 chromatin accessibility by depositing the H4K20me1 mark. Notably, SETD8 inhibition reduced H4K20me1 levels on the HSV-1 genome.

### SETD8 inhibition reduced the levels of elongating viral mRNAs

To circumvent heterochromatic suppression, HCF-1 promotes multiple stages of IE gene transcription through interactions with transcription factors, epigenetic modifiers, and components of the SEC (19, 20, 24, 26, 29, 30, 33, 34). It has been established that SEC-mediated transcriptional elongation is essential for optimal IE gene expression, and inhibition of the SEC suppresses HSV-1 lytic infection and reactivation from latency (33, 34). Critically, the SEC promotes the release of RNAPII from its paused state located 30–50 bp downstream of the TSS to enable transcriptional elongation and production of full-length IE mRNAs.

**Fig 7:**
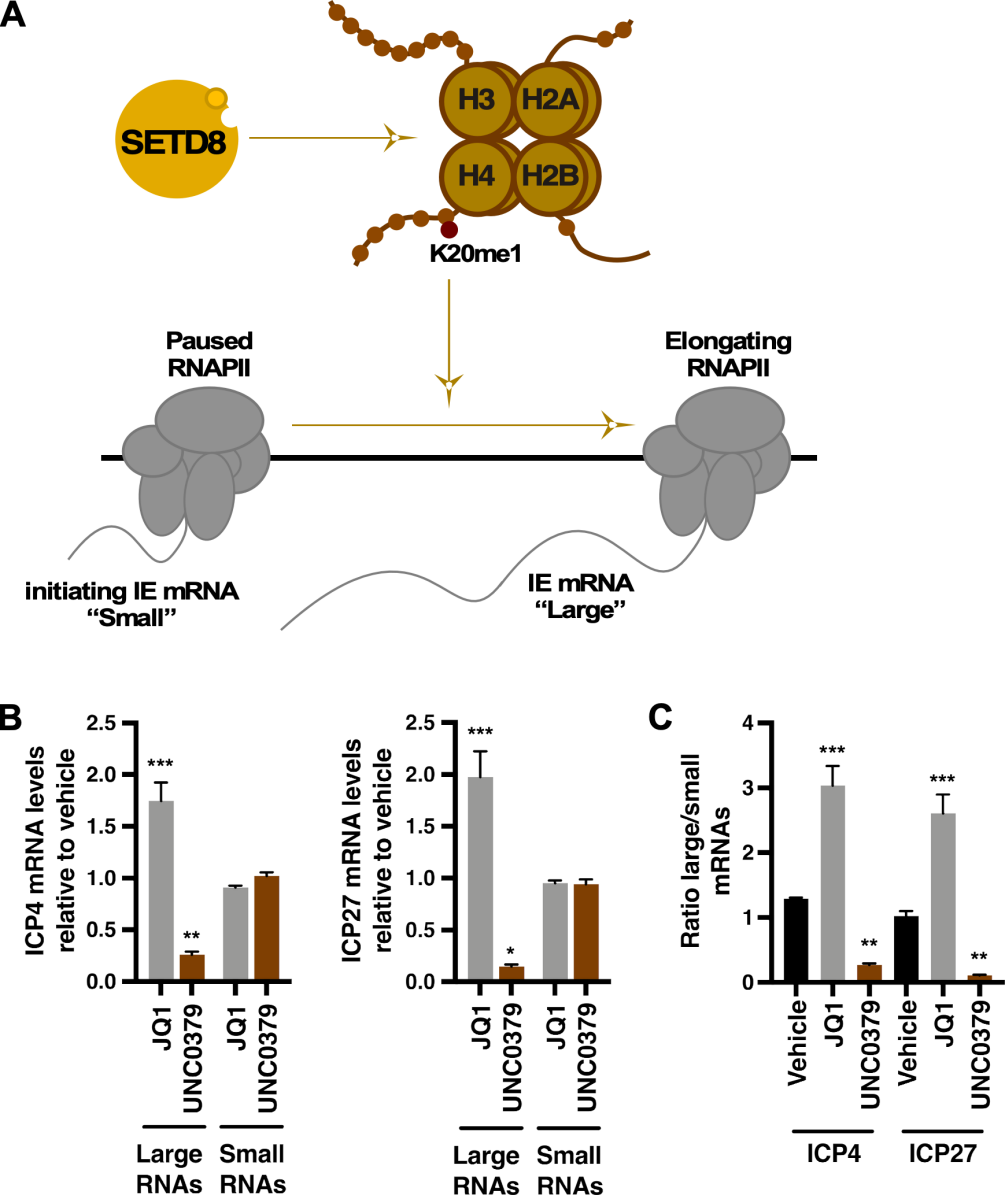
UNC0379 suppressed transcriptional elongation of IE mRNAs. (**A**) Model of SETD8 regulation of transcriptional elongation of HSV-1 IE genes. SETD8-mediated deposition of H4K20me1 results in the release of paused RNAPII (initiating “Small” IE mRNAs) to transcriptional elongation (“Large” IE mRNA). (**B, C**) Levels of large and initiating small viral IE (ICP4 and ICP27) mRNAs in cells treated with vehicle, 2 µM JQ1, or 4 µM UNC0379 and infected with HSV-1 (MOI 1) for 1.5 h. mRNA levels are shown relative to those in cells treated with vehicle and as a (**C**) ratio of large/small mRNA. Data are from three experiments (≥ 9 replicate samples). *, *P* < 0.05; **, *P* < 0.01; ***, *P* < 0.001.

In addition to modulating chromatin accessibility, SETD8 promotes transcriptional elongation of cellular genes (40, 41). The H4K20me1 modification that is deposited by SETD8 recruits the MSL/MOF histone acetylase complex. The resulting histone acetylation increases nucleosome mobility along the DNA fiber, facilitating RNAPII elongation. Therefore, to determine if SETD8 was required for HSV-1 IE transcriptional elongation, we blocked the activity of SETD8 with UNC0379 and performed an assay that measured the quantity of “large” elongating viral mRNA (which is indicative of transcriptional elongation), compared to that of the “small” initiating mRNAs (Fig. 7A) (34). For ICP4 and ICP27, there was no impact on the quantity of initiating small RNAs with SETD8 inhibition (Fig. 7B). The positive control JQ1, a selective inhibitor of BET family of bromodomains previously shown to stimulate transcriptional elongation of HSV-1 and HIV (34, 51–53), increased the large HSV-1 IE mRNA population. In contrast, UNC0379 treatment suppressed transcriptional elongation, as indicated by decreased proportions of large IE mRNA. Expressed as a ratio of the large IE mRNAs to small mRNAs, SETD8 inhibition significantly reduced the relative abundance of large IE transcripts (Fig. 7C). These results suggest SETD8 mediated both chromatin accessibility and transcription elongation of HSV-1 IE genes during lytic infection.

### The initiation of HSV-1 reactivation from latency was suppressed with SETD8 inhibition

**Fig 8:**
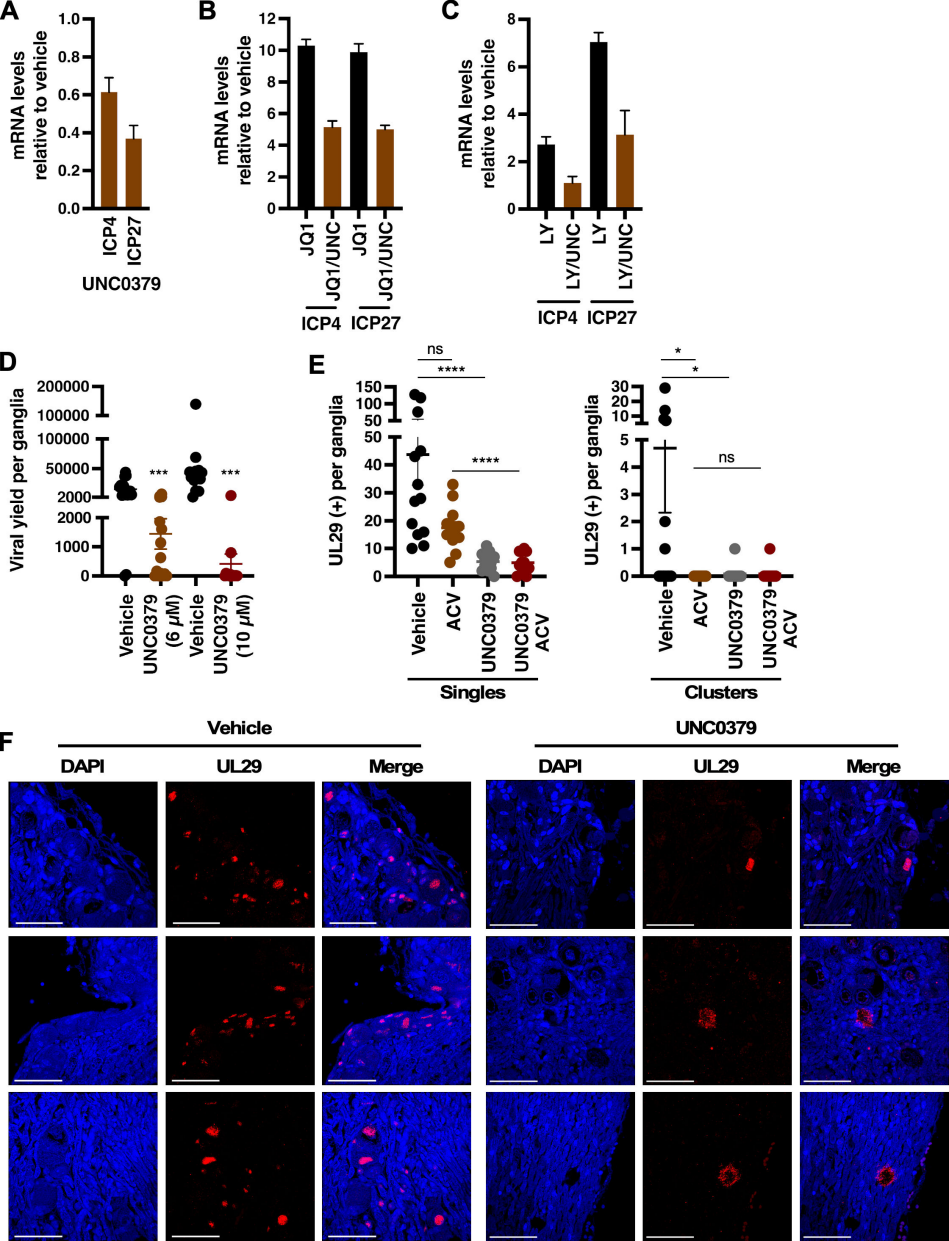
UNC0379 suppressed HSV-1 latency-reactivation in a mouse ganglia explant model. (**A–C**) mRNA levels of viral IE (ICP4 and ICP27) in latently infected trigeminal ganglia explanted in the presence of vehicle or specified inhibitor for 12 h. Data are from two experiments (4 pools of 5 ganglia per condition). (**A**) Ganglia explanted in the presence of vehicle or 10 µM UNC0379. (**B**) Ganglia explanted in the presence of vehicle, JQ1 (1 µM), or JQ1 and UNC0379 (UNC, 6 µM). (**C**) Ganglia explanted in the presence of vehicle, LY294002 (LY, 20 µM), or LY294002 and UNC0379 (UNC, 6 µM). (**D**) Viral yields from latently infected trigeminal ganglia explanted in the presence of vehicle or UNC0379 (6 or 10 µM) for 48 h. Data are yields (PFU) from individual ganglia; *n* ≥ 14 per group and displayed as PFU/ganglia. (**E, F**) Latently infected trigeminal ganglia explanted in the presence of vehicle, ACV (100 µM), UNC0379 (10 µM), or UNC0379 and ACV for 48 h. Sections were stained for UL29 (HSV-1 lytic ssDNA binding protein; red) and DAPI (blue). (**E**) The numbers of individual UL29(+) neurons per ganglia (left panel) and UL29(+) neuron clusters [right panel, >3 adjacent UL29(+) neurons]; *n* ≥ 12 ganglia per group. (**F**) Representative images from three different ganglia; scale bar, 50 µm. *, *P* < 0.05; ***, *P* < 0.001; ****, *P* < 0.0001; ns, not significant.

Given the potent suppression of HSV-1 lytic infection by UNC0379 (Fig. 3; Fig. S1 and S2), we next assessed whether SETD8 inhibition would block latency reactivation. To this end, we utilized the mouse model of HSV-1 latency reactivation and infected the eyes of Balb/C mice with HSV-1. The clearance of primary infection and establishment of viral latency in sensory neurons occurs ~40 days post-infection (dpi) (54). To induce a robust reactivation, trigeminal ganglia were explanted into culture media and monitored for HSV-1 reactivation by measurement of viral IE mRNAs (Fig. 8A through C), viral yields (Fig. 8D), and the expression of the lytic viral protein UL29 (Fig. 8E and F).

Transcription of representative IE genes (ICP4 and ICP27) was reduced in ganglia explanted in the presence of UNC0379 (Fig. 8A). Previous studies showed that the BET inhibitor JQ1 and PI3K inhibitor LY294002 accelerated HSV-1 reactivation in ganglia explants (34, 55, 56). To further evaluate the requirement of SETD8 for the transcription of IE genes during reactivation, ganglia were explanted in the presence of either vehicle, JQ1, LY294002, or combinations of JQ1 or LY294002 with UNC0379 (Fig. 8B and C). Explanting with JQ1 or LY294002 increased IE transcript levels. Importantly, explant in the presence of SETD8 inhibitor UNC0379 suppressed IE gene transcription, even under accelerated reactivation conditions with JQ1 or LY294002. Given the reliance on IE gene expression for production of progeny virus during reactivation, we also observed a significant decrease in viral yields when determined by plaque assays (Fig. 8D). Thus, SETD8 inhibition suppressed both IE transcription and viral yields during reactivation.

To directly assess the number of neurons undergoing primary reactivation, ganglia were explanted in the presence of vehicle, ACV, UNC0379, or the combination of UNC0379 and ACV (Fig. 8E). Ganglia tissue sections were stained with an antibody that recognizes the lytic viral protein UL29 to identify those neurons undergoing reactivation (Fig. 8F). Quantitation of the number of UL29-positive [UL29(+)] single neurons, representing the primary reactivation event, revealed a significant decrease with UNC0379 treatment as compared to vehicle control (Fig. 8E). Under physiological (*in vivo*) conditions, HSV-1 reactivation does not spread laterally through the ganglia; however, in the ganglia explant model, axotomy and explantation serve as recurrent reactivation triggers, and lateral spread is readily observed at 48 h following explantation (26, 35, 57, 58). To distinguish between effects on primary reactivation versus viral spread (clusters) in this model, ACV was included to block viral spread. Compared to ACV alone, there was a significant decrease in the number of UL29(+) single neurons in ganglia explanted in the combination of UNC0379 and ACV. Furthermore, SETD8 inhibition also suppressed reactivation spread, as measured by reduced UL29(+) clusters. Collectively, we conclude that SETD8 was required for the initiation of primary HSV-1 reactivation from latency.

### SETD8 inhibition suppressed primary HSV-1 ocular infection *in vivo*

**Fig 9:**
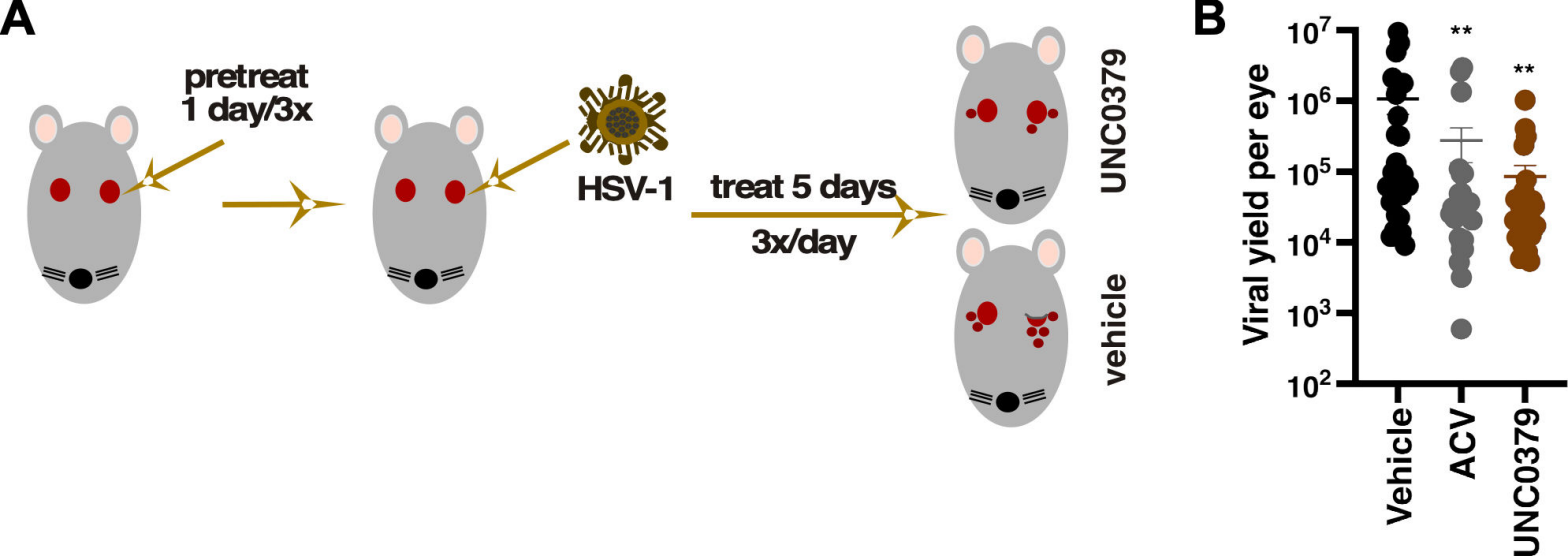
An inhibitor of SETD8 reduced primary ocular infection *in vivo*. (**A**) *In vivo* infection experimental design. Mice were pretreated ocularly with vehicle, 200 µM ACV, or 50 µM UNC0379 followed by ocular infection with HSV-1 (5 × 10^5 PFU/eye). Treatment with compounds continued three times daily. (**B**) Viral yields were determined from eyes collected at 5 dpi; *n* ≥ 28 eyes and displayed as PFU/eye. **, *P* < 0.01.

The chemical probe UNC0379 revealed the importance of SETD8 in the initiation of HSV-1 reactivation from latency in the ganglia explant model and during *in vitro* lytic infection of fibroblast cells. To assess the effect of UNC0379 during primary *in vivo* infections, the eyes of Balb/C mice were pretreated topically with vehicle, 200 µM ACV, or 50 µM UNC0379 1 day prior to ocular HSV-1 infection (Fig. 9A). Eyes were continuously treated three times daily until 5 dpi. As indicated in Fig. 9B, UNC0379 treatment significantly reduced the progression of ocular infection, as measured by a decrease in viral titers of ~1 log compared to the vehicle. Results demonstrated that topical application of the epigenetic inhibitor UNC0379 was as effective as ACV in reducing ocular titers, with no observable adverse effects on the eye. These findings support further investigation of SETD8 inhibition as an alternative or combinatorial antiviral therapy to HSV-1 infections.

## DISCUSSION

The lytic replication cycle, latency, and reactivation stages of HSV-1 are tightly coupled to the epigenetic machinery of the infected host cell (15, 16, 18–20, 23–28, 30–32, 34–37, 55). Importantly, the lytic replication cycle consists of a highly ordered transcriptional program that depends on the expression of IE genes. The IE proteins have functions not only in stimulating the sequential wave of E genes, genome amplification, and L genes but also in counteracting host responses to infection. These functions include altering the host splicing machinery, restricting mRNA transport, and promoting ubiquitin-mediated degradation of immune mediators (2). Significantly, disruption of IE gene expression leads to defects in lytic replication and reactivation from latency (21–26, 31–35, 55, 58).

HSV-1 genome accessibility is a key determinant for establishing the lytic replication program (15, 16, 18, 23). This is important because, immediately after nuclear entry, nucleosomes assembled on the HSV-1 genome are marked by repressive heterochromatin signatures (H3K9me3 and H3K27me2) (24–28). This initial cell-mediated repression is counteracted by the HCF-1 coactivator complex as it proceeds through interactions with multiple protein complexes. HCF-1 activity is potentiated by recruitment of transcription factors, H3K9-demethylases (LSD1 and JMJD2), and H3K4-methyltransferases (SETD1A and MLL1) to HSV-1 promoter/enhancer regions, thereby stimulating IE transcription (19, 20, 24, 26, 29, 30, 32, 35, 36). Inhibition of either LSD1 or JMJD2 results in enhanced epigenetic repression of the HSV-1 genome, blocking both lytic infection and latency reactivation (24, 26, 32). While elements of the HCF-1 coactivator complex have been defined, the epigenetic components and mechanisms regulating HSV-1 chromatin at IE genes are not completely understood. To identify additional epigenetic factors that regulate IE gene transcription, we screened a curated chemical library of 48 epigenetic inhibitors. We identified several epigenetic inhibitors that modulated HSV-1 IE gene transcription. Among these, the chemical probe UNC0379 targeting the H4K20 mono-methyltransferase SETD8 effectively repressed IE gene transcription.

Epigenetic inhibitors have previously been utilized as chemical probes to interrogate the stages of HSV-1 lytic infection and latency reactivation (19, 20, 24, 26, 32, 55, 56, 58). In this study, treatment of human fibroblast cells with the SETD8 inhibitor UNC0379 significantly repressed HSV-1 IE gene expression across a wide range of MOIs, reduced viral yields, and limited the spread of lytic infection. Moreover, UNC0379 treatment displayed no impact on viral entry and no indication of cytotoxicity at the evaluated concentration, thus supporting the premise that the disruption of IE gene transcription was specifically targeted. We also found the initiation of HSV-1 reactivation was suppressed in latently infected mouse sensory ganglia. Most significantly, *in vivo* topical application of the SETD8 inhibitor suppressed HSV-1 titers in a mouse ocular model. The data presented here support the critical role of SETD8 in regulating HSV-1 IE gene expression during lytic infection and the initiation of primary reactivation from latency.

**Fig 10:**
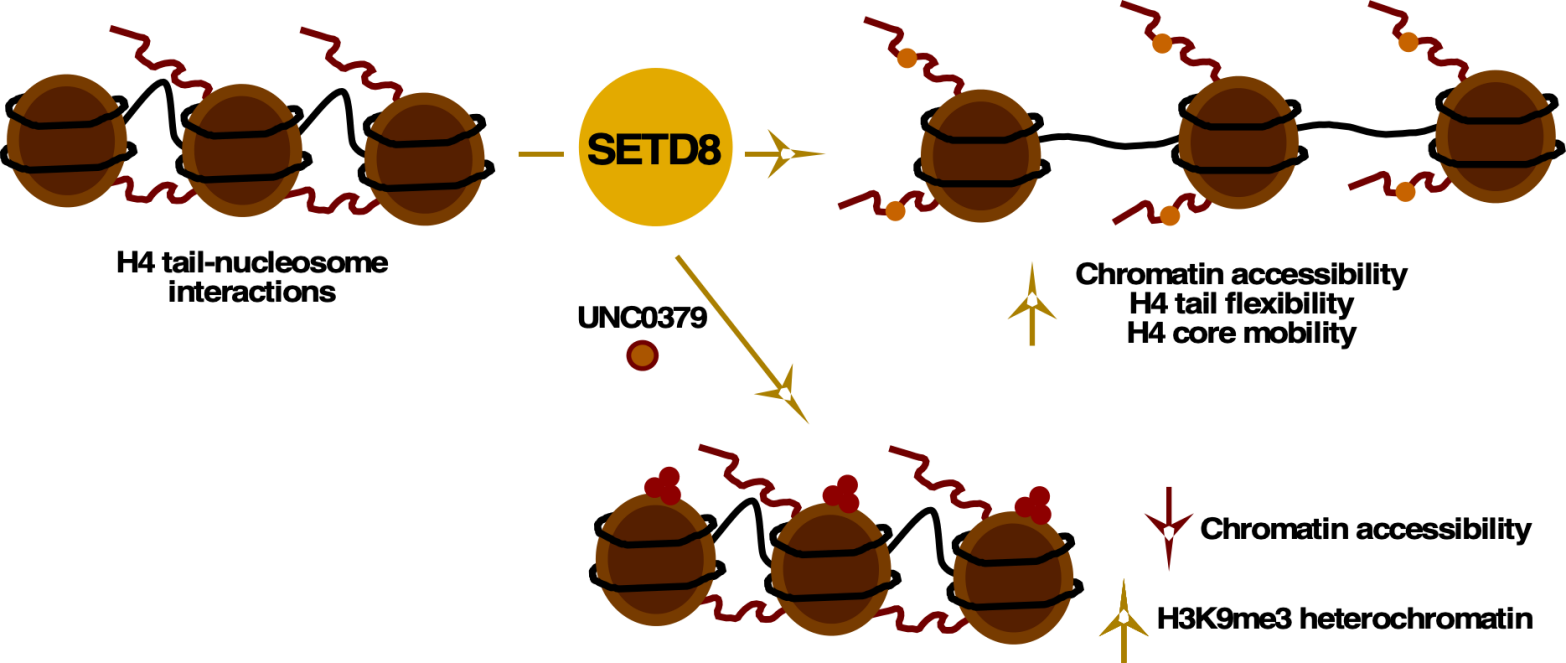
Model of SETD8-mediated modulation of HSV-1 gene expression. SETD8-mediated mono-methylation promotes H4 tail flexibility, H4 core mobility, and an increase in inter-nucleosomal distance to enhance gene expression (39). UNC0379 inhibition of SETD8 results in reduced chromatin accessibility and increased heterochromatin associated with the HSV-1 genome. UNC0379 effectively suppressed HSV-1 IE gene expression, lytic infection, and viral reactivation from latency.

One mechanism by which SETD8 promotes cellular gene expression is through H4K20-monomethylation, which leads to increased chromatin accessibility (39). The H4K20me1 modification enhances the flexibility of the H4 tail, resulting in a greater inter-nucleosomal distance and elevated histone mobility. In the context of HSV-1, we hypothesized that SETD8 facilitates the dynamics of chromatin modulation during the early events of IE gene transcription by promoting HSV-1 genome accessibility (Fig. 10). This hypothesis was supported by ChIP-QPCR assays, which showed increased H3K9me3 heterochromatin and reduced HCF-1 and RNAPII occupancy at IE genes following UNC0379 treatment. This repression was not restricted to the IE gene cluster as ChIP-seq illustrated a global decrease in RNAPII binding across the HSV-1 genome. Mechanistically, reduced H4K20me1 levels correlated with a genome-wide decrease in HSV-1 chromatin accessibility, as determined by ATAC-seq. These results suggest that decreased H4K20me1 deposition at viral IE genes leads to reduced recruitment of HCF-1 and RNAPII, likely due to diminished chromatin accessibility. While HCF-1 does not directly bind DNA, it is plausible that decreased chromatin accessibility also impairs the recruitment of transcription factors (such as VP16, OCT-1, Sp1, and GABP) that recruit HCF-1 to IE promoter regions. It is also important to note that unlike the cellular genome, the HSV-1 genome is initially assembled into unstable nucleosome structures that are highly accessible to MNase digestion or to Tn5 transposase insertion in ATAC-seq assays (15–18, 59, 60). Accordingly, we hypothesize that the high ATAC-seq coverage of shallow peaks observed in the vehicle group (Fig. 5A) reflects the overall high level of chromatin accessibility resulting from these unstable nucleosomes, particularly during the early phase of infection (1.5 hpi).

With SETD8 inhibition, the decrease in H4K20me1 levels observed by ChIP-QPCR (Fig. 6, 1 hpi) appeared to be restricted to the HSV-1 genome, with no change detected in a representative cellular control. To determine whether UNC0379 exhibits selectivity for HSV-1 chromatin over the cellular chromatin, we assessed total H4K20me1 levels in cells treated with UNC0379. Western blot analysis of acid-extracted histones revealed that prolonged UNC0379 treatment (24 and 48 h) reduced total H4K20me1 levels, while no significant change was observed at 5 h (Fig. S6). These results suggest that global changes in H4K20me1 levels on cellular chromatin require extended treatment. It is tempting to speculate that HSV-1 nucleosome assembly may involve the incorporation of histones initially free of the H4K20me1 modification, followed by the active recruitment of SETD8 to deposit H4K20me1 on the viral genome early during infection. In contrast, cellular chromatin contains histones already marked with H4K20me1 and thus requires extended UNC0379 treatment to inhibit SETD8 activity and potentially promote turnover of H4K20me1 by the demethylase PHF8/KDM7B. Further investigation into the kinetics of H4K20me1 deposition and removal on HSV-1 and cellular chromatin remains an important area of future interest.

In addition to H4K20me1-mediated regulation of chromatin accessibility, our results suggest SETD8 controls HSV-1 replication on multiple levels, including transcriptional elongation. Under SETD8 inhibition, we observed reduced levels of large elongating IE mRNAs, suggesting a block in the release of paused RNAPII or a reduced rate of transcriptional elongation. Untangling the specific function(s) of SETD8 in transcriptional elongation of IE genes is beyond the scope of this study. However, these results are supported by evidence that H4K20me1 recruits histone acetyltransferase MSL/MOF to cellular genes, which promotes the release of paused RNAPII (40, 41). In parallel, it has been clearly shown that transcription elongation of HSV-1 IE genes is essential for optimal lytic infection and reactivation (33, 34).

As an additional layer of complexity, a previous study by Chen et al. found that UNC0379 treatment of THP-1 cells suppressed HSV-1 genome amplification at 10 hpi (61). Mechanistically, SETD8-mediated methylation of PCNA was proposed to be required for its stability, thereby suppressing HSV-1 DNA amplification upon UNC0379 treatment (61, 62). Results from our study demonstrated a decrease in IE transcription with UNC0379 treatment as early as 1.5 hpi (Fig. 3A). Based on the kinetics of the lytic gene cascade, we anticipate that UNC0379-mediated suppression of IE genes would also lead to a decrease in HSV-1 genome amplification at later times post-infection. Furthermore, we observed that UNC0379-mediated reduction of H4K20me1 levels on the HSV-1 genome is an early event (1 hpi, Fig. 6), while Chen et al. reported no change during longer periods of infection (10 hpi) (61). Collectively, these findings suggest SETD8 targets multiple stages of the viral replication cycle (chromatin accessibility, transcriptional elongation, and genome amplification), making it a promising druggable target to suppress HSV-1 infection.

 In addition to SETD8, our epigenetic inhibitor screen identified chemical probes targeting protein arginine methyltransferase 1 (PRMT1; TC-E5003) (42), PRMT3 (SGC-707) (43), PRMT4 (TP-064) (44), and Type I PRMTs (MS023; PRMT1, -3, -4, -6, and -8) (45), all of which suppressed HSV-1 IE transcription (Fig. 2; Table S2). A previous study has shown that arginine methylation of ICP27 disrupts its stability and localization, resulting in defects in HSV-1 transcription and viral titers (63). In another study, HSV-1 infection was reduced in heterozygous PRMT3 mice and after *in vivo* treatment with the PRMT3 inhibitor SGC-707 (64). Mechanistically, it was found that PRMT3-mediated methylation of DNA and RNA sensors cGAS, RIG-I, and MDA5 attenuates their activation during HSV-1 infection. The corollary of our epigenetic inhibitor screen highlights the need for further investigation into the roles of PRMT1, PRMT3, and PRMT4 in the HSV-1 replication cycle, and by extension, to other human herpesviruses.

In this study, we screened a curated epigenetic library containing 48 compounds to identify novel factors involved in HSV-1 IE gene transcription during lytic infection. We identified the H4K20 mono-methyltransferase SETD8 as a critical factor for promoting viral IE gene expression during lytic infection and the initiation of viral reactivation from latency. Significantly, topical application of the SETD8 inhibitor UNC0379 suppressed primary ocular infection of mice *in vivo*. Our working model proposes that SETD8-mediated H4K20 mono-methylation increases HSV-1 chromatin accessibility, thereby facilitating recruitment of the HCF-1 transcription coactivator complex and RNAPII (Fig. 10). This study demonstrated that UNC0379 treatment increased repressive H3K9me3 heterochromatin and decreased both chromatin accessibility and H4K20me1 levels. The inhibition of SETD8 also reduced the fraction of elongating HSV-1 IE mRNAs. Importantly, UNC0379 treatment also suppressed HCMV transcription, suggesting a common regulatory mechanism among herpesviruses. Because UNC0379 inhibited HSV-1 IE transcription across a wide range of MOIs, it is tempting to speculate that further research is warranted to evaluate UNC0379 as a potential combination therapy with ACV, particularly in cases of ACV resistance, as observed in the immunocompromised population or during prolonged treatment for recurrent HSV ocular infections (11–14, 32). Taken together, these results indicate that SETD8 is an essential epigenetic modulator of HSV-1 chromatin and represents a promising novel antiviral therapeutic target.

## MATERIALS AND METHODS

### Cell cultures and viral infections

Telomerase-immortalized HFF cells (TERT-human foreskin fibroblasts), MRC-5 cells, and Vero cells were maintained according to standard protocols. Viral infections with HSV-1 (Strain 17) and human cytomegalovirus (HCMV, Towne strain) were performed by infecting cells at the indicated multiplicity of infection (MOI) in Dulbecco’s modified Eagle medium (DMEM) containing 1% fetal bovine serum (FBS) and 1% penicillin/streptomycin for 1 h at room temperature (RT). Following absorption, the inoculum was removed and then the cells were washed with PBS to remove unbound virus and then incubated for the indicated duration with DMEM containing 1% FBS at 37°C/5% CO_2_. Where specified, viral yields were determined by plaque assay on Vero cells using limiting dilution of homogenized cells or mouse ganglia or eyes.

### Epigenetic inhibitor screen

Epigenetic inhibitors were obtained from the NCATS or commercial sources (Table S1). Unless otherwise stated, HFF cells were pretreated for 4 h with a vehicle control (dimethyl sulfoxide, DMSO) or the indicated inhibitor. Treated cells were infected with HSV-1 (MOI 1) at RT for 1 h in the absence of inhibitor, followed by continued infection in the presence of inhibitor for 1.5 h at 37°C. The BET bromodomain inhibitor JQ1(+) and the JMJD2 inhibitor ML324 served as positive controls (26, 34).

### Cell viability assays

HFF cells were treated with vehicle or the indicated concentrations of UNC0379, NSC663284, or saponin (cytotoxic control) for 5.5 h. Cell viability was measured using a BioTek Synergy Neo2 Hybrid Multimode Reader and the CellQuanti-MTT Kit (BioAssay Systems) according to the manufacturer’s instructions.

### Animals and infections

#### Viral reactivation in explants of sensory ganglia

Female 8-week-old BALB/cAnNTac (Taconic Biosciences) mice were infected ocularly with 5 × 10^6^ PFU of HSV-1 (strain F). Following the establishment of latency (~40 dpi), mice were randomized, trigeminal ganglia were bisected, and paired halves were incubated in media containing control vehicle or UNC0379. Viral yields were determined at 48 h post explant by titering homogenates of ganglia on Vero cell monolayers. mRNA levels of viral and control cellular genes were determined after explant of latently infected ganglia in the presence of vehicle, UNC0379, JQ1, LY294002, or a combination of JQ1/UNC0379 or LY294002/UNC0379 for 12 h. For quantitation of neurons undergoing viral reactivation, ganglia were explanted for 48 h in the presence of vehicle, UNC0379, ACV, or UNC0379/ACV. Ganglia were subsequently fixed in 4% paraformaldehyde for 12 h and embedded in paraffin (54). Deparaffinized sections were treated with citric acid for antigen retrieval, stained with anti-UL29 antibody, and visualized using a Leica TCS-SP8 laser scanning confocal microscope.

#### Primary ocular HSV-1 infection

BALB/cAnNTac mice were pretreated ocularly by topical application (5 µL per eye in 0.1% methylcellulose) of 50 µM UNC0379, 200 µM ACV, or vehicle, followed by ocular infection with 5 × 10^5^ PFU of HSV-1 (Strain F) per eye. Treatments were continued three times per day, and eyes were harvested at 5 dpi for determination of viral yields.

### RNA isolation and quantitation

For quantitation of mRNA from cultured cells, RNA was isolated using the NucleoSpin RNA Isolation Kit (Macherey Nagel), RNA concentrations were determined with a spectrophotometer (DeNovix, DS-11FX+), and cDNAs were synthesized using qScript cDNA SuperMix (Quanta Biosciences). Specific cDNAs were quantitated in triplicate by RT-QPCR using SYBR green master mix (Roche) on a QuantStudio 3 (Applied Biosystems; QuantStudio v1.5.3 software). Isolation of individual small (<200 nucleotides) and large (>200 nucleotides) RNA fractions was performed as previously described with minor modifications (34). Briefly, HFF cells were lysed in TriPure Isolation Reagent (Sigma Aldrich), followed by the addition of chloroform according to the manufacturer’s instructions. Total RNA in the aqueous phase was recovered with ethanol added to a final concentration of 35% and applied to RNA isolation columns (Macherey Nagel). Following centrifugation, the column-bound large RNA fraction was isolated according to the manufacturer’s instructions, while the final ethanol concentration of the unbound small RNA fraction was increased to 70% and applied to a second RNA isolation column. cDNAs from small RNA fractions were prepared using the qScript microRNA cDNA Synthesis kit (Quanta Biosciences), while the large RNA fraction was reverse-transcribed using qScript cDNA SuperMix (Quanta Biosciences). For quantitation of mRNAs from trigeminal ganglia, pools of five ganglia were lysed in TriPure Isolation Reagent (Sigma Aldrich) and disrupted using Lysing Matrix D (MP Biomedicals) in a FastPrep-24 bead beating grinder (6.0 m/s, 40 sec cycles, three cycles), and RNA was isolated using the NucleoSpin RNA Isolation Kit (Macherey Nagel). Trigeminal ganglia samples were normalized to mRNA levels of murine GAPDH. Primer sequences are listed in Table S3.

### Immunoblotting

HFF cells were pretreated with vehicle or 4 µM and 8 µM of either UNC0379 or NSC663284 and then infected with HSV-1 (MOI 2) for 3 h. Cells were lysed in radioimmunoprecipitation assay (RIPA) buffer [50 mM Tris-HCl (pH 7.5), 250 mM NaCl, 1 mM EDTA, 1% NP40, 1% sodium deoxycholate, 0.1% SDS, complete protease inhibitors, 2 mM NaV0_4_, 1 mM NaF, and 10 mM B-glycerophosphate] and briefly agitated with a QSonica Q500 Cup Sonicator (amplitude 75%, 3 cycles of 30 s on/off, 4°C). For acid-extracted histones, cells were resuspended in Triton extraction buffer [TEB (0.5% Triton X-100, PBS, complete protease inhibitors)] and lysed on ice for 10 m. Following centrifugation (6,500*×g* for 10 m, 4°C) and a second wash with TEB, nuclei were resuspended in 0.2 N HCl and incubated overnight at 4°C. Acid-extracted histones were collected from the supernatant after centrifugation. Protein extracts were resolved by SDS-PAGE and analyzed by Western blot using the antibodies listed in Table S3. Blots were visualized with WesternBright Quantum (Advansta) and quantitated with a G:BOX Chemi XT4 (Syngene; GeneTools 4.3.17.0 v software).

### Impact of UNC0379 on HSV-1 entry/transport

HFF cells were pretreated with vehicle or 2 µM and 4 µM UNC0379 and then infected with HSV-1 (MOI 1) for 1.5 h. Total cellular DNA was isolated with the Quick-DNA Miniprep Plus kit (Zymo Research). For the nuclear fraction, HFF cells were resuspended in 0.4 mL buffer A (10 mM HEPES pH 7.9, 10 mM KCl, and 1.5 mM MgCl_2_) for 15 min on ice, and then the cell membrane was lysed with 25 µL 10% NP40. After vortexing and centrifugation, DNA from nuclear cell pellets was isolated with the Quick-DNA Miniprep Plus kit (Zymo Research). Total and nuclear DNAs were quantitated in triplicate by RT-QPCR. Viral DNA levels were determined using primers to the viral UL30 gene, and samples were normalized to the levels of the cellular GAPDH gene.

### Chromatin immunoprecipitation (ChIP) and ChIP-seq assays

ChIP assays were done essentially as described (35) with the following modifications. Isolated nuclei were lysed, and samples were sonicated using a Covaris M220 Focused-Ultrasonicator (8 m, 10% duty cycle, 75 W incident power, 200 burst per cycle) to obtain DNA fragments of 200–500 base pairs (bp). Chromatin samples (350–450 µg) were precleared with Protein G Dynabeads (ThermoFisher) for 1 h at 4°C followed by incubation with 3–5 µg antibodies for 14 h at 4°C. Antibodies used are listed in Table S3. For ChIP-seq, chromatin samples were incubated with 1:1,000 dilution of anti-RNAPII-NTD (Cell Signaling 14958), and library construction was prepared by the CCR-Sequencing Facility, Frederick National Laboratory for Cancer Research. Libraries were sequenced on a NovaSeq X Plus 1.5B to yield 3.10–3.60 × 10^8^ paired-end reads per sample.

### Assay for transposase-accessible chromatin sequencing (ATAC-seq) sample processing

HFF cells were pretreated with vehicle or 4 µM UNC0379 and infected with HSV-1 (MOI 5) for 1.5 h. Nuclear extracts, DNA transposition, and library construction were prepared by the CCR-Sequencing Facility, Frederick National Laboratory for Cancer Research according to Caravaca et al. (65). Libraries were sequenced on a NovaSeq X Plus 1.5B to yield 2.16–2.56 × 10^8^ paired-end reads per sample.

### ChIP-seq and ATAC-seq analysis

Reads were processed using our chrom-seek (1.2.0) pipeline (https://github.com/OpenOmics/chrom-seek). Reads were trimmed with Cutadapt version 4.4 (66) and then aligned to a custom reference genome composed of the human hg38 genome plus the HSV-1 reference chromosome NC_001806.2 using BWA version 0.7.17 (67). All reads aligning to the Encode hg38 blacklist regions (68) were identified and removed with Picard SamToFastq (https://broadinstitute.github.io/picard/). Reads with a mapQ score less than 6 were removed with SAMtools version 1.17 (69), and PCR duplicates were removed with Picard MarkDuplicates. Data were converted into bigwig files for viewing and normalized by reads per genomic content (RPGC) of the combined reference genome using deepTools version 3.5.1 (70). For ChIP-seq, each sample was subtraction-corrected against its input control. Coverage plots were created using karyoploteR and GenomicRanges. This work utilized the computational resources of the NIH HPC Biowulf cluster (http://hpc.nih.gov).

### Confocal microscopy

Immunofluorescent microscopy was done according to standard protocols using antibodies listed in Table S3. Stained trigeminal ganglia sections were visualized with a Leica TCS-SP8 laser scanning confocal microscope using a 63X oil immersion objective. Deconvolution was completed with Huygens Essential (Scientific Volume Imaging; 24.04.0p7), and sequential z-sections were assembled with Imaris software (Bitplane AG, 10.0.1).

### Statistical analyses

Results are presented as mean±standard error of the means (SEM). Analyses were done using Prism (GraphPad 10.4.1). Further details are provided in the figure legends and in Table S4. Asterisks indicate significance as follows: *, *P* < 0.05; **, *P* < 0.01; ***, *P* < 0.001; ****, *P* < 0.0001; ns, not significant.

## Data Availability

The ATAC-seq and RNAPII ChIP-seq data presented in the study are openly available in the Gene Expression Omnibus at https://www.ncbi.nlm.nih.gov/geo/ under GSE302606 and GSE302607. All remaining data sets are available in the main text and supplemental material.

## SUPPLEMENTAL MATERIAL

Supplemental material jvi.01293-25-s0001.pdf Figures S1 to S6; Tables S1 to S4.

ASM does not own the copyrights to Supplemental Material that may be linked to, or accessed through, an article. The authors have granted ASM a non-exclusive, world-wide license to publish the Supplemental Material files. Please contact the corresponding author directly for reuse.
