# Supplementary material for: The H4K20-mono-methyltransferase SETD8 promotes global accessibility of infecting herpes simplex virus genomes
Source: J Virol. 2025 Nov 19;99(12):e01293-25. doi: 10.1128/jvi.01293-25 (PMC12724366; doi:10.1128/jvi.01293-25)
Supplement: Supplemental material — Figures S1 to S6; Tables S1 to S4. [file jvi.01293-25-s0001.pdf]

SUPPLEMENTAL MATERIAL

The H4K20-mono-methyltransferase SETD8 Promotes Global Accessibility of Infecting Herpes Simplex Virus Genomes

Jesse H. Arbuckle, Andy A. Yanez, Syeda S. Baksh, Tovah E. Markowitz, Jodi L. Vogel, Alison A. McBride, Thomas M. Kristie

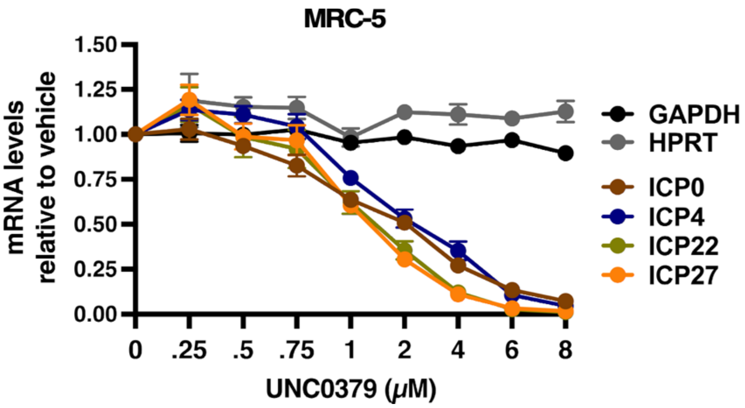

IE mRNA levels	UNC0379 IC50 (μM)
**ICP0**	1.822
**ICP4**	2.426
**ICP22**	1.092
**ICP27**	0.957

Supplemental Figure 1. UNC0379 suppressed HSV-1 IE gene expression in MRC-5 cells. MRC-5 cells were treated with the indicated concentrations of UNC0379 and infected with HSV-1 (MOI 1). mRNA levels of viral IE (ICP0, ICP4, ICP22, ICP27) and cellular control genes (GAPDH, HPRT) are shown relative to levels in vehicle treated cells at 1.5 hpi. Data are from 3 experiments (4 - 6 replicate samples). The IC50 values for each transcript are shown in the table to the right.

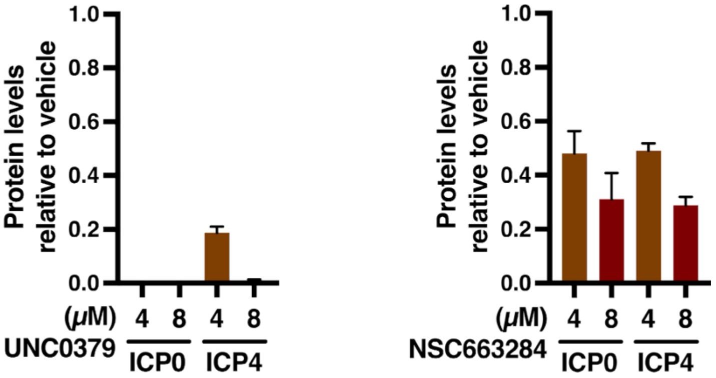

Supplemental Figure 2. Inhibition of SETD8 reduced IE protein levels. Densitometry analysis of western blots (Figure 3C) for viral IE (ICP0, ICP4) and cellular (actin for normalization) proteins in HFF cells treated with vehicle, 4 *µ*M or 8 *µ*M UNC0379, or NSC663284 and infected with HSV-1 (MOI 2) for 3 h. Data are from 3 experiments (6 replicates).

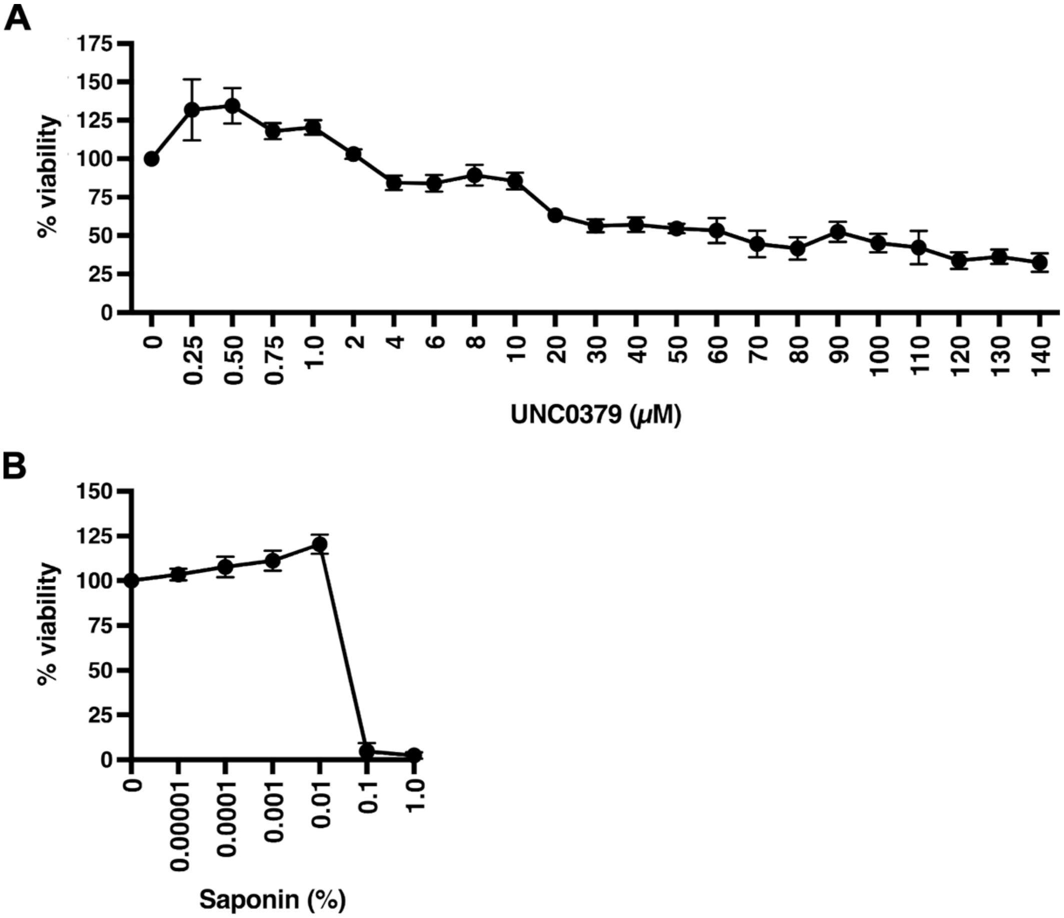

Supplemental Figure 3. Cell viability assays. HFF cells were treated with the indicated concentrations of (**A**) UNC0379 or (**B**) Saponin (cytotoxic control) for 5.5 h. Cell viability was determined by MTT assays. CC_{50} for UNC0379 was determined to be 62.6 μM. Data are from 4 experiments (12-13 replicate samples).

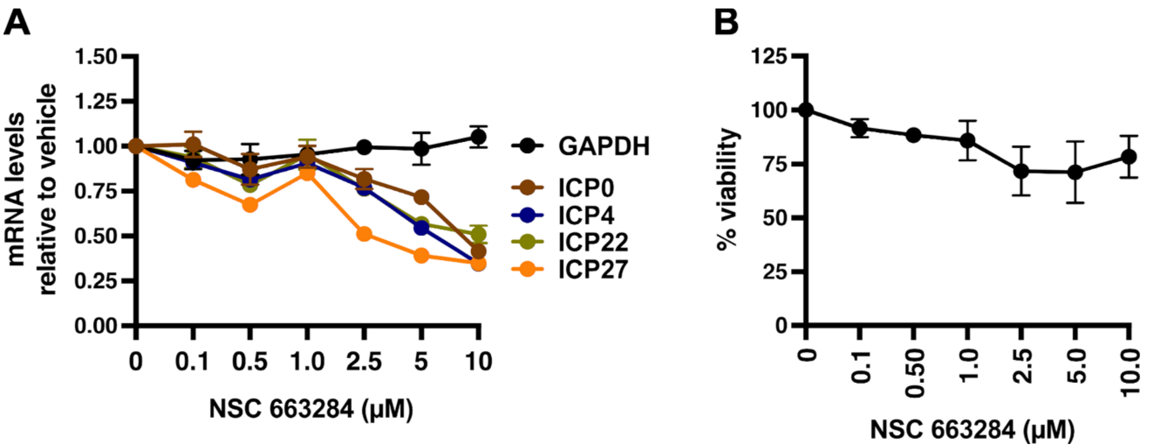

Supplemental Figure 4. NSC663284 suppressed IE gene expression. (**A**) HFF cells were treated with the indicated concentrations of NSC663284 and infected with HSV-1 (MOI 1) for 1.5 h. mRNA levels of viral IE genes (ICP0, ICP4, ICP22, ICP27) and cellular control GAPDH are shown relative to levels in vehicle treated cells. Data are from 3 experiments (6 replicate samples). (**B**) HFF cells were treated with the indicated concentrations of NSC663284 for 5.5 h. Cell viability was determined by MTT assays (4 replicate samples).

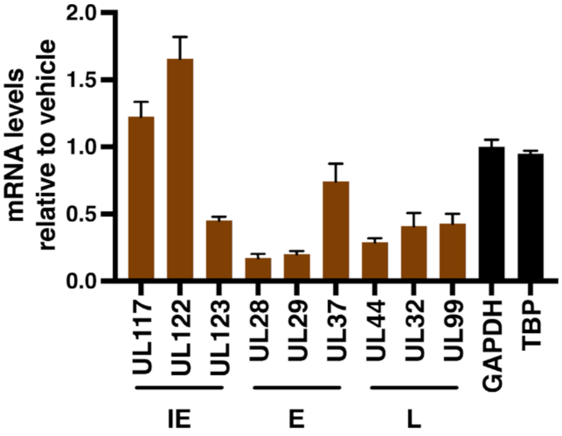

Supplemental Figure 5. HCMV gene expression is suppressed with SETD8 inhibition. HFF cells were treated with 8 μM UNC0379 and infected with HCMV (MOI 0.2) for 4 h. mRNA levels of HCMV (IE = UL117, UL122, UL123; E = UL28, UL29, UL37; L = UL44, UL32, UL99) (1) and cellular control genes (GAPDH, TBP) are shown relative to levels in vehicle treated cells. Data are from 3 experiments.

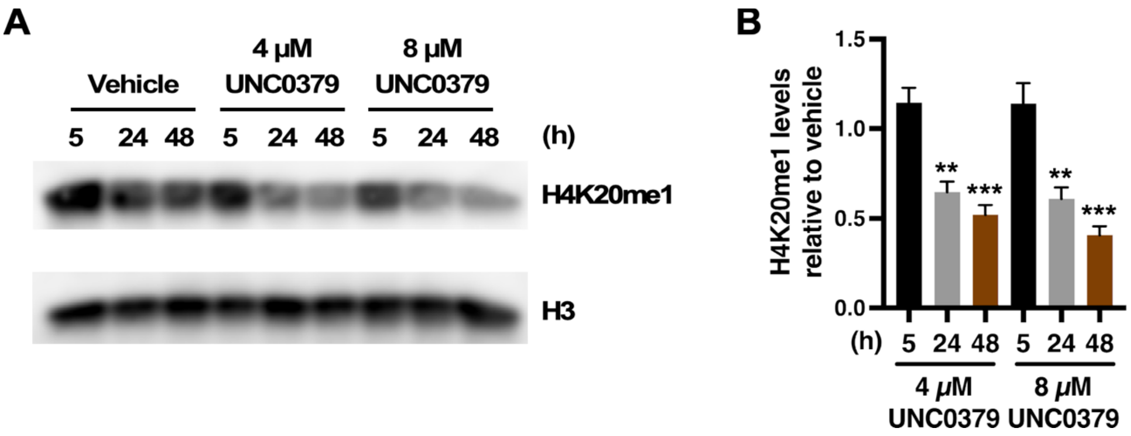

Supplemental Figure 6. Inhibition of SETD8 reduced H4K20me1 protein levels.
HFF cells were treated with vehicle, 4 *μ*M, or 8 *μ*M UNC0379 for 5, 24, and 48 h. (**A**) Representative western blot of acid extracted histones probed for H4K20me1 and H3. (**B**) Densitometry analysis of western blots for H4K20me1 levels, normalized to H3. Data are from 5 experiments.

INHIBITORS	***Source***
A-196	NCATS: NCGC00387310-01; MedChemExpress: HY-100201
A-366	NCATS: NCGC00351478-04; Cayman Chemical: 16081
ACV	Sigma Aldrich: A4669
AZ505	NCATS: NCGC00378958-02
BAY-598	Selleckchem: E7480
BAY-6035	Cayman Chemical: 25925
BI-9564	NCATS: NCGC00481108-01
CPI-455	Cayman Chemical: 22127
EPZ-004777	NCATS: NCGC00356803-03; Cayman Chemical: 16173
EPZ-015666	NCATS: NCGC00386436-03; Cayman Chemical: 17285
EPZ-020411	NCATS: NCGC00482383-01; Cayman Chemical: 19160
EPZ-5676	NCATS: NCGC00381557-02
GSK5959	NCATS: NCGC00107508-04
GSK591	Cayman Chemical: 18354
GSK602	NCATS: NCGC00387308-01
GSK6853	Cayman Chemical: 20985; Tocris: 6198
GSK9311	Tocris: 6203
JQ1(+)	APExBIO:A1910
KDM5-C70	NCATS: KDM5-C70
KDM5A-IN-1	NCATS: NCGC00482457-01; MedChemExpress: HY-100014
KDOAM-25	NCATS: NCGC00388700-01; MedChemExpress: HY-102047B
LLY-283	Cayman Chemical: 21596
LLY-284	Sigma Aldrich: SML1971
LY294002	Cayman Chemical: 70920
MI-2	NCATS: NCGC00345491-08; Cayman Chemical: 29218
ML324	Selleckchem: S7296
MM-102	NCATS: NCGC00351775-04; Cayman Chemical: 17699
MS023	NCATS: NCGC00483169-01; Cayman Chemical: 34786
MS049	NCATS: NCGC00480748-01
NI 57	NCATS: NCGC00483115-01; Cayman Chemical: 17662
NSC663284	Cayman Chemical: 13303
TC-E5003	NCATS: NCGC00370996-03
OICR-9429	NCATS: NCGC00371263-02
PFI-3	NCATS: NCGC00371001-05; Cayman Chemical: 15267
PFI-4	NCATS: NCGC00476204-01
SGC OF-1	NCATS: NCGC00384195-05
SGC-0946	NCATS: NCGC00344622-10
SGC-707	Cayman Chemical: 17017
TC-E 5002	NCATS: NCGC00373154-02
Thiamet G	NCATS: NCGC00344096-02
TP-064	Tocris: 6008; Cayman Chemical: 20256
TP-472	Tocris: 6000; Cayman Chemical: 200030
TP-472N	Sigma Aldrich: SML1933; Tocris: 5999
UNC926	NCATS: NCGC00379146-01
UNC0638	NCATS: NCGC00189220-12
UNC1215	NCATS: NCGC00344623-12
UNC669	NCATS: NCGC00347957-09; Cayman Chemical: 10875
UNC0379	NCATS: NCGC00384189-06; Sigma Aldrich: 1620401-83-3; Cayman Chemical: 16400
UNC0642	NCATS: NCGC00189140-03; Cayman Chemical: 14604
UNC1079	Cayman Chemical: 20566
WDR5 0103	NCATS: NCGC00384201-02

Supplemental Table 1. List of epigenetic inhibitors and source. Tabular list of epigenetic inhibitors with their sources that were used in this study.

ICP27 IE mRNA levels relative to Vehicle

Inhibitor	Target	0.01 μM	0.05 μM	0.1 μM	0.25 μM	0.5 μM	1.0 μM	2.5 μM	4 μM	5 μM	10 μM	20 μM	25 μM	50 μM
BI-9564	BRD9			0.99 (0.02)			0.80 (0.05)				1.02 (0.12)			
GSK602	BRD9/7			1.21 (0.09)			0.95 (0.34)				1.07 (0.05)			
TP-472	BRD9/7			0.96 (0.15)			0.95 (0.16)							
TP-472N	Control for TP-472			0.92 (0.15)			1.09 (0.05)							
RATIO 472/472N				1.04			0.87							
GSK5959	BRPF1			0.78 (0.20)			0.78 (0.11)					0.92 (0.04)		
PFI-4	BRPF1				0.77 (0.06)		0.89 (0.06)		0.95 (0.01)					
SGC OF-1	BRPF1				0.86 (0.01)		1.00 (0.13)		1.28 (0.12)					
NI 57	BRPF1/2/3		1.20 (0.02)	1.15 (0.14)			1.23 (0.03)	1.04 (0.03)		0.85 (0.01)	1.17 (0.02)			
GSK6853	BRPF1	1.18 (0.10)	1.13 (0.09)	1.33 (0.05)			1.01 (0.02)			0.94 (0.06)				
GSK9311	Control for GSK6853	0.98 (0.06)		0.99 (0.04)			0.91 (0.02)							
RATIO 6853/9311		1.20		1.34			1.11							
EPZ-5676	DOT1L			1.19 (0.06)			1.14 (0.10)			1.27 (0.09)				
SGC-0946	DOT1L			1.16 (0.19)			1.26 (0.30)			0.98 (0.01)				
EPZ-004777	DOT1L			1.31 (0.02)			0.99 (0.12)			0.95 (0.05)				
A-366	G9a/GLP	1.17 (0.10)	1.17 (0.14)	1.15 (0.08)		1.22 (0.02)	0.89 (0.11)			0.76 (0.03)	0.74 (0.05)			
UNC0638	G9a/GLP						1.15 (0.11)			1.04 (0.00)	0.93 (0.22)			
UNC0642	G9a/GLP			1.25 (0.08)			1.19 (0.03)	1.37 (0.01)		0.89 (0.06)				
UNC0379	SETD8 (KMT5A)						0.64 (0.05)			0.19 (0.03)	0.06 (0.01)		0.01 (0.00)	
AZ505	SMYD2			1.00 (0.12)			1.03 (0.20)				1.13 (0.21)			
BAY-598	SMYD2						1.16 (0.20)				1.15 (0.08)		1.23 (0.07)	
BAY-6035	SMYD3	0.83 (0.01)	0.78 (0.05)	0.79 (0.05)			0.93 (0.05)				0.91 (0.13)		0.75 (0.11)	
A-196	SUV420H1/2						0.95 (0.04)			1.01 (0.20)	1.02 (0.05)		0.76 (0.02)	
TC-E5002	KDM2/7						1.07 (0.17)				0.88 (0.02)		0.96 (0.07)	
KDOAM-25	KDM5A/B/C/D						0.78 (0.00)				0.77 (0.02)		0.70 (0.03)	
KDM5A-IN-1	KDM5A/B/C	0.80 (0.04)	0.82 (0.06)			1.17 (0.14)	1.67 (0.23)			0.9 (0.09)	0.75 (0.02)		0.71 (0.06)	
KDM5-C70	KDM5A/B						1.18 (0.09)	1.33 (0.02)		0.86 (0.06)	0.71 (0.07)		0.86 (0.03)	
CPI-455	KDM5A	1.06 (0.02)	0.99 (0.02)	1.10 (0.02)		0.76 (0.06)	0.54 (0.03)			0.61 (0.00)	0.69 (0.00)		0.69 (0.00)	
UNC926	L3MBTL1						0.78 (0.06)			0.95 (0.10)			0.69 (0.05)	
UNC669	L3MBTL1						1.04 (0.06)			1.15 (0.08)			1.00 (0.02)	
UNC1215	L3MBTL3			0.82 (0.07)			0.84 (0.16)				0.72 (0.03)		0.79 (0.06)	
UNC1079	Control for UNC1215			0.96 (0.19)			0.85 (0.04)				0.79 (0.06)		0.92 (0.13)	
RATIO 1215/1079				0.85			0.99				0.91		0.86	
MM-102	MLL	1.08 (0.00)	1.07 (0.05)	1.26 (0.00)		0.77 (0.01)	0.67 (0.06)			0.90 (0.01)	0.97 (0.12)			
MI-2	MLL/MENIN	0.80 (0.03)	0.93 (0.09)	1.61 (0.25)		1.05 (0.03)	1.21 (0.21)			0.85 (0.03)	0.65 (0.04)			
OICR-9429	MLL/WDR5			0.95 (0.13)			0.86 (0.12)				1.23 (0.46)		1.32 (0.07)	
WDR5 0103	MLL1/ASH2L/RBBP5/WDR5						1.00 (0.04)				0.83 (0.13)		1.09 (0.16)	
Thiamet G	O-GlcNAcase						0.88 (0.08)				0.86 (0.12)		0.77 (0.14)	
TC-E5003	PRMT1			0.81 (0.06)			0.71 (0.04)				0.62 (0.05)			
MS023	PRMT1/3/4/6/8			0.82 (0.04)			0.60 (0.03)				0.63 (0.08)			
SGC-707	PRMT3			0.75 (0.09)			0.62 (0.05)				0.11 (0.00)			
MS049	PRMT4/6			1.17 (0.14)			1.06 (0.03)				1.17 (0.39)			
TP-064	PRTM4	0.78 (0.00)	0.77 (0.02)	0.74 (0.04)			0.72 (0.06)			0.68 (0.06)	0.57 (0.06)	0.39 (0.03)		
EPZ015666	PRMT5	1.02 (0.02)	0.94 (0.06)	1.07 (0.14)		0.76 (0.08)	0.60 (0.03)			0.74 (0.08)	0.81 (0.06)			
GSK591	PRMT5			1.02 (0.00)			0.69 (0.12)				0.83 (0.07)	0.77 (0.10)	0.78 (0.04)	
LLY-283	PRMT5			0.63 (0.09)			0.88 (0.23)				0.72 (0.03)			
LLY-284	Control for LLY-283			0.82 (0.17)			0.72 (0.16)				0.75 (0.20)			
RATIO 283/284				0.77			1.22				0.96			
EPZ020411	PRMT6			0.86 (0.20)			0.81 (0.08)				0.74 (0.16)			
PFI-3	SMARCA2/4			1.00 (0.29)			0.95 (0.00)				0.73 (0.06)			
JQ1(+)	BET bromodomain						2.52 (0.69)							
ML324	JMJD2													0.01 (0.01)

Supplemental Table 2. Titration of epigenetic inhibitors identified compounds suppressing HSV-1 IE gene expression. HFF cells were pretreated for 4 h with the indicated inhibitors and infected with HSV-1 (MOI 1) for 1.5 h. mRNA levels of the viral IE ICP27 are relative to those in vehicle treated cells and are normalized to the cellular control SP1 or GAPDH. Data are from ≥ 2 replicate samples. The standard deviation for each sample is indicated in parenthesis. Blank cells were untested concentrations.

PRIMERS TARGET	***SEQUENCE***
	qRT-PCR
ICP0 (HSV-1)	CCCACTATCAGGTACACCAGCTT<br>CTGCGCTGCGACACCTT<br>*Liang (2013) mBio 7:4*
ICP4 (HSV-1)	GAAGTTGTGGACTGGGAAGG<br>GTTGCCGTTTATTGCGTCTT<br>*Ottosen (2006) Virology 345:468*
ICP22 (HSV-1)	TTTGGGGAGTTTGACTGGAC<br>CAGACACTTGCGGTCTTCTG<br>*Ottosen (2006) Virology 345:468*
ICP27 (HSV-1)	GCATCCTTCGTGTTTGTCATTCTG<br>GCATCTTCTCTCCGACCCCG<br>*Arbuckle (2017) mBio 8:4*
UL117 (HCMV)	TCGTAGAAAAGCAGCCAGAC<br>AGGCGAAAGCTGGTGAAT
UL122 (HCMV)	ATGGTTTTGCAGGCTTTGATG<br>ACCTGCCCTTCACGATTCC<br>*Cristea (2010) J Virol 84:15*
UL123 (HCMV)	GCCTTCCCTAAGACCACCAAT<br>ATTTTCTGGGCATAAGCCATAATC<br>*Mitchel (2009) J Virol 83:19*
UL28 (HCMV)	GGATGGATGGAACCCGTGAACA<br>ACGAAACCAGAAGGAGCCCTGAC<br>*Mitchel (2009) J Virol 83:19*
UL29 (HCMV)	CCGATGCTCTCGTAGCGAAAGTC<br>GCTGGTGGGGCAGGATAAGTTG<br>*Mitchel (2009) J Virol 83:19*
UL37 (HCMV)	GACGAAGTCCGATGAGGAGGATG<br>TGGGACACTGGGCGTTGTTG<br>*Liang (2013) mBio 7:4*
UL44 (HCMV)	GCCCGATTTCAATATGGAGTTCAG<br>CGGCCGAATTCTCGCTTTC<br>*Terhune (2010) PLoS Pathog 6:6*
UL32 (HCMV)	GGTTTCTGGCTCGTGGATGTCG<br>CACACAACACCGTCGTCCGATTAC<br>*Mitchel (2009) J Virol 83:19*
UL99 (HCMV)	GTGTCCCATTCCCGACTCG<br>TTCACAACGTCCACCCACC<br>*Mitchel (2009) J Virol 83:19*
SP1 (Human)	TCAGAACCCACAAGCCCAAAC<br>TGCCAGCAGGAATGGAAGC<br>*Liang (2013) mBio 7:4*
TBP (Human)	TGACCCCCATCACTCCTGC<br>CGTGGTTCGTGGCTCTCTTATC<br>*Liang (2013) mBio 7:4*
HPRT (Human)	ATTGTAATGACCAGTCAACAGGG<br>GCATTGTTTTGCCAGTGTCAA<br>*Alfonso-Dunn (2020) mBio 11:3*
GAPDH (Human)	TTCGACAGTCAGCCGCATCTTCTT<br>CAGGCGCCCAATACGACCAAATC<br>*Cliffe (2008) J Virol 82:12030*
GAPDH (Mouse)	CTGACGTGCCGCCTGGAGAAA<br>CCCGGCATCGAAGGTGGAAGAGT<br>*Arbuckle (2017) mBio 8:4*
	DNA qPCR
UL30 (HSV-1)	AGAGGGACATCCAGGACTTTGT<br>CAGGCGCTTGTTGGTGTAC<br>*Hill (2014) Sci Transl Med 6:265*
	SHORT RNA AMPLIFICATION
ICP4 Promoter-Proximal (+57) Forward	GCCAGAGACAGACCGTCAGA<br>*Alfonso-Dunn (2017) Cell Host Microbe 21:4*
ICP27 Promoter-Proximal (+81) Forward	CCACCACCAGAGGCCATATC<br>*Alfonso-Dunn (2017) Cell Host Microbe 21:4*

PRIMERS TARGET	***SEQUENCE***
	ChIP qPCR
ICP0 (-293 to -150)	TTCGGGAAGGCGGGAAG<br>CCCAAAGAATATCATTAGCATGCAC
ICP0 (+242 to +380)	ATGTCTGGGGCGCCATATT<br>GTTCCACGCCCCCTAACAT
ICP0 (+3086 to +3226)	ACAGCCTCTGGATGACCCC<br>TTGTTTTCCCTCGTCCCG<br>*Arbuckle (2014) mBio 5:1*
ICP4 (-374 to -245)	GCGGGGCTCGTATCTCATTA<br>CCGCATGGCATCTCATTACC
ICP4 (+57 to +153)	GCCAGAGACAGACCGTCAGA<br>ATGCTTGGGTGGGAAAAAGG
ICP4 (+3822 to +3957)	GAAGTTGTGGACTGGGAAGG<br>ATCAGGTTGTTGCCGTTTATTG
UL29 (-69 to +3)	CCTTTTGTCAATCGGTCCGC<br>CGGGAGACATACCTTGTCGG<br>*Cohen (2018) PLoS Pathog*
UL29 (+2098 to +2178)	GGGCGCAACTTTCGCAATCAATTC<br>GGCCGACAGAAACCCGTTGTTAAA
UL44 (-244 to -166)	CGCCGGTGTGTGATGATTT<br>TTTATACCCGGGCCCCAT<br>*Cohen (2018) PLoS Pathog*
GAPDH (-144 to +21)	TACTAGCGGTTTTACGGGCG<br>TCGAACAGGAGGAGCAGAGAGCGA<br>*MilliporeSigma*
ZNF554	CGGGGAAAAGCCCTATAAAT<br>TCCACATTCACTGCATTCGT<br>*Frietze (2010) PLoS One 5:12*
RPL5	AACAGGGCTTTAAGATTGTTCTG<br>CACTTCCTAAAGGTCTTTCATGC<br>*Tanaka (2017) Cell Rep 18*
REAGENTS	
NucleoSpin RNA	Macherey Nagel: 740955.250
qScript cDNA Synthesis Kit	Quanta Biosciences: 95047
Maxima First Strand cDNA Synthesis Kit	ThermoScientific: K1672
Quanta qScript microRNA cDNA Synthesis Kit	Quanta Biosciences: 95107
TriPure Isolation Reagent	Sigma Aldrich: 11667165001
Lysing Matrix D	MP Biomedicals: 6913100
Antigen Unmasking Solution, Citric Acid Based	Vector Laboratories: H-3300
WesternBright Quantum HRP substrate	Advansta: K-12042-D20
cOmplete protease inhibitors	Roche: 05056489001
ChIP DNA Clean & Concentrator	Zymo Research: D5205
Dynabeads Protein G	Invitrogen: 10004D
Quick-DNA Miniprep Plus Kit	Zymo Research: D4069
DMSO	Sigma Aldrich: D2650
CellQuanti-MTT Kit	BioAssay Systems: CQMT-500
Saponin	BioAssay Systems: CTTX-050
**ANTIBODIES AND CELLULAR MARKERS**	
ICP4 (clone 10F1)	Virusys: H1A021
ICP0	Santa Cruz: sc-56985
Actin	Santa Cruz: sc-47778
HCF-1 (Ab2124)	Kristie Lab; Kristie (1995) J Biol Chem
HCF-1 (Ab2130)	Kristie Lab; Kristie (1995) J Biol Chem
RNAPII	Cell Signaling: 14958
H3 total	Abcam: ab1791
H3K9me3	Abcam: ab8898
H4K20me1	Active motif: 39027, ABclonal: A2370
UL29	Gift of W. Ruyechan (SUNY Buffalo)
Donkey anti-rabbit IgG-Alexa 594	Jackson ImmunoResearch: 711-585-152
ProLong Glass Antifade Mountant with NucBlue	Invitrogen: P36985
VIRUSES	
HSV-1 (Strain 17)	Gift of N. Fraser, University of Pennsylvania
HSV-1 (Strain F)	Gift of B. Roizman, University of Chicago
hCMV (TB40/E-gfp)	Gift of S. Terhune, Medical College of Wisconsin
CELL LINES	
Tert-HFF	Gift of T. Shenk, Princeton University
MRC-5	ATCC: CCL-171
Vero	ATCC: CCL-81

Supplemental Table 3. Reagents and primers. Reagents and primer sequences used in this study.

Figure	Panel	Experiment	Description	Test	Comparison	*p1*	*p2*	Experiments/samples	*n*
**2**		Epigenetic inhibitor screen	ICP27 and control cellular mRNA levels in HFF cells treated with Vehicle or indicated inhibitor					2-4 replicate samples	n = 2, 3, or 4
**3**	**A, B**	UNC0379 titration in HFF cells	HSV-1 and control cellular mRNA levels in HFF cells treated with Vehicle or UNC0379	ANOVA with Dunnett's multiple comparisons test	ICP0 mRNA levels; Vehicle vs 0.25-8 μM UNC0379<br>ICP4 mRNA levels; Vehicle vs 0.25-8 μM UNC0379<br>ICP22 mRNA levels; Vehicle vs 0.25-8 μM UNC0379<br>ICP27 mRNA levels; Vehicle vs 0.25-8 μM UNC0379<br>SP1 mRNA levels; Vehicle vs 0.25-8 μM UNC0379<br>TBP mRNA levels; Vehicle vs 0.25-8 μM UNC0379	2 μM; 0.0008 ***<br>1 μM; 0.0453 *<br>2 μM; 0.0143 *<br>2 μM; 0.0249 *<br>8 μM; 0.7952 ns<br>8 μM; >0.9999 ns	4 μM; <0.0001 ****<br>2 μM; <0.0001 ****<br>4 μM; 0.0008 ***<br>4 μM; <0.0001 ****	3 experiments; each 2 replicate samples	6<br>6<br>4<br>6<br>6<br>6
**3**	**C**	Western blot	HSV-1 and cellular protein levels in HFF cells treated with Vehicle, UNC0379 or NSC663284					3 experiment; 4-6 replicate samples	6
**3**	**D**	UNC0379 impact on nuclear accumulation of viral DNA	Viral DNA levels (Total and Nuclear) in HFF cells treated with Vehicle or UNC0379	ANOVA with Dunnett's multiple comparisons	Viral DNA levels; Total cellular; Vehicle vs 2-4 μM UNC0379<br>Viral DNA levels; Nuclear; Vehicle vs 2-4 μM UNC0379	0.9903 ns<br>0.6347 ns		3 experiments; each 3 replicate samples	3<br>3
**3**	**E**	Time of addition	HSV-1 mRNA levels in HFF cells treated with Vehicle or UNC0379 at various times relative to HSV-1 infection	ANOVA with Dunnett's multiple comparisons test	ICP0 mRNA levels; Vehicle vs UNC0379 addition -4 to 1.5 hpi<br>ICP4 mRNA levels; Vehicle vs UNC0379 addition -4 to 1.5 hpi<br>ICP22 mRNA levels; Vehicle vs UNC0379 addition -4 to 1.5 hpi<br>ICP27 mRNA levels; Vehicle vs UNC0379 addition -4 to 1.5 hpi	1.0 h; <0.0001 ****<br>1.0 h; <0.0001 ****<br>1.0 h; <0.0001 ****<br>1.0 h; <0.0001 ****		3 experiments; each >/= 8 replicate samples	Vehicle n = 9; UNC0379 n = 8
**3**	**F**	MOI dependence	HSV-1 mRNA levels in HFF cells infected with various MOIs and treated with Vehicle or UNC0379	ANOVA with Dunnett's multiple comparisons test	ICP0 mRNA levels; MOI 1 vs 2-5-10 in presence of UNC0379<br>ICP4 mRNA levels; MOI 1 vs 2-5-10 in presence of UNC0379<br>ICP22 mRNA levels; MOI 1 vs 2-5-10 in presence of UNC0379<br>ICP27 mRNA levels; MOI 1 vs 2-5-10 in presence of UNC0379	MOI 5; 0.4602 ns<br>MOI 5; 0.9827 ns<br>MOI 5; 0.9832 ns<br>MOI 5; 0.8990 ns	MOI 10; 0.4237 ns<br>MOI 10; 0.9417 ns<br>MOI 10; 0.8333 ns<br>MOI 10; 0.9996 ns	3 experiments; 8 replicate samples	3
**3**	**G**	Viral yields-one step growth	Viral yields (PFU/well) from HFF cells infected for 12 h in the presence of Vehicle or UNC0379	ANOVA with Dunnett's multiple comparisons test	Viral DNA levels; Vehicle vs 100 μM ACV<br>Viral DNA levels; Vehicle vs 4 μM UNC0379<br>Viral DNA levels; Vehicle vs 8 μM UNC0379	0.0029 **<br>0.0036 **<br>0.0029 **		4 experiments; each 3 replicates	4
**3**	**H**	Viral yields-spread assay	Viral yields (PFU/well) from HFF cells infected for 8 h followed by addition of Vehicle or UNC0379 for 12 h	Unpaired two-tailed t test	Viral DNA levels; Vehicle vs UNC0379, MOI 0.01<br>Viral DNA levels; Vehicle vs UNC0379, MOI 0.1	0.0001 ***<br>0.0018 **		3 experiments; each 2-3 replicate samples	Vehicle n = 7; UNC0379 n = 8
**4**	**A**	HCF	ChIP assays; HCF-1 occupancy at viral IE promoters in HFF cells treated with Vehicle or UNC0379					3 experiments	3
**4**	**B**	RNAPII	ChIP assays; RNAPII occupancy at viral IE regions in HFF cells treated with Vehicle or UNC0379	Unpaired two-tailed t test	Ratio of levels at ICP0 promoter vs GAPDH control: Vehicle vs UNC0379<br>Ratio of levels at ICP4 promoter vs GAPDH control: Vehicle vs UNC0379	0.0031 **<br>0.0035 **		3 experiments	3
**4**	**C**	H3, H3K9me3	Levels of H3, H3K9me3 at viral promoters in HFF cells treated with Vehicle or UNC0379	Unpaired two-tailed t test	Ratio levels of H3 at ICP0 promoter vs ZNF554 control: Vehicle vs UNC0379<br>Ratio levels of H3K9me3 at ICP0 promoter vs ZNF554 control: Vehicle vs UNC0379<br>Ratio levels of H3 at ICP4 promoter vs ZNF554 control: Vehicle vs UNC0379<br>Ratio levels of H3K9me3 at ICP4 promoter vs ZNF554 control: Vehicle vs UNC0379<br>Ratio levels of H3 at UL29 promoter vs ZNF554 control: Vehicle vs UNC0379<br>Ratio levels of H3K9me3 at UL29 promoter vs ZNF554 control: Vehicle vs UNC0379<br>Ratio levels of H3 at UL44 promoter vs ZNF554 control: Vehicle vs UNC0379<br>Ratio levels of H3K9me3 at UL44 promoter vs ZNF554 control: Vehicle vs UNC0379	0.0009 ***<br>0.0132 *<br>0.016 *<br>0.0009 ***<br>0.0004 ***<br>0.0043 **<br>0.0044 **<br>0.0004 ***		3 experiments	3
**4**	**D, E**	ChIP-Seq	RNAPII ChIP-Seq mean RPGC for HFF cells treated with Vehicle or UNC0379					2 experiments	2
**5**	**A, B**	ATAC-Seq	ATAC-Seq mean RPGC for HFF cells treated with Vehicle or UNC0379	One-tailed t-test	Vehicle vs UNC0379	0.0006 ***		3 experiments	3
**6**		H4K20me1	ChIP assays; H4K20me1 occupancy at viral IE and E regions in HFF cells treated with Vehicle or UNC0379					3 experiments	3
**7**	**B, C**	Large/small mRNAs	Large and small HSV-1 mRNAs from HFF cells treated with Vehicle, JQ1 or UNC0379	ANOVA with Dunnett's multiple comparisons test	Large ICP4 mRNAs; Vehicle vs JQ1<br>Large ICP4 mRNAs; Vehicle vs UNC0379<br>Small ICP4 mRNAs; Vehicle vs JQ1<br>Small ICP4 mRNAs; Vehicle vs UNC0379<br>Ratio large/small ICP4 mRNAs; Vehicle vs JQ1<br>Ratio large/small ICP4 mRNAs; Vehicle vs UNC0379<br><br>Large ICP27 mRNAs; Vehicle vs JQ1<br>Large ICP27 mRNAs; Vehicle vs UNC0379<br>Small ICP27 mRNAs; Vehicle vs JQ1<br>Small ICP27 mRNAs; Vehicle vs UNC0379<br>Ratio large/small ICP27 mRNAs; Vehicle vs JQ1<br>Ratio large/small ICP27 mRNAs; Vehicle vs UNC0379	0.0004 ***<br>0.0049 **<br>0.6134 ns<br>0.9354 ns<br>0.0002 ***<br>0.0036 **<br><br>0.0003 ***<br>0.0116 *<br>0.4498 ns<br>0.2603 ns<br>0.0004 ***<br>0.0059 **		3 experiments; each 3-4 replicate samples	Vehicle n = 9; JQ1 n = 9; UNC0379 n = 12
**8**	**A**	Ganglia explant mRNAs	HSV-1 mRNAs from latently infected ganglia explanted for 12 h in Vehicle or UNC0379					2 experiment; 2 pools of 5 ganglia	2
**8**	**B**	Ganglia explant mRNAs	HSV-1 mRNAs from latently infected ganglia explanted for 12 h in Vehicle, JQ1 or JQ1/UNC0379					2 experiment; 2 pools of 5 ganglia	2
**8**	**C**	Ganglia explant mRNAs	HSV-1 mRNAs from latently infected ganglia explanted for 12 h in Vehicle, LY294002, or LY294002/UNC0379					2 experiment; 2 pools of 5 ganglia	2
**8**	**D**	Ganglia explant viral yields	Viral yields (PFU/ganglia) from latently infected ganglia explanted for 48 h in Vehicle or UNC0379	Wilcoxon matched-pairs signed rank test	Viral yields; Vehicle vs 6 μM UNC0379<br>Viral yields; Vehicle vs 10 μM UNC0379	0.0009 ***<br>0.0002 ***			6 μM UNC0379 n = 15<br>10 μM UNC0379 n = 14
**8**	**E**	Ganglia explant UL29+ neurons	UL29+ neurons in ganglia explanted for 48 h in Vehicle, ACV, UNC0379, or UNC0379/ACV	Mann Whitney test	UL29+ single neurons; Vehicle vs UNC0379<br>UL29+ single neurons; Vehicle vs ACV<br>UL29+ single neurons; ACV vs UNC0379/ACV<br>UL29+ neuron clusters; Vehicle vs UNC0379<br>UL29+ neuron clusters; Vehicle vs ACV<br>UL29+ neuron clusters; ACV vs UNC0379/ACV	<0.0001 ****<br>0.0504 ns<br><0.0001 ****<br>0.0353 *<br>0.0149 *<br>>0.9999 ns			Vehicle n = 13; UNC0379 n = 12<br>Vehicle n = 13; ACV n = 12<br>n = 12<br>Vehicle n = 13; UNC0379 n = 12<br>Vehicle n = 13; ACV n = 12<br>n = 12
**9**		In vivo ocular infection	Viral yields from eyes of mice infected with HSV-1 and treated with Vehicle or UNC0379 for 5 d	Kruskal-Wallis test	Vehicle vs ACV<br>Vehicle vs UNC0379	0.0100 **<br>0.0011 **			Vehicle n = 28; ACV n = 28; UNC0379 n = 30
**S1**		UNC0379 titration in MRC-5 cells	Viral mRNA levels in MRC-5 cells treated with Vehicle or UNC0379					3 experiment; 4-6 replicate samples	6
**S2**		Western blot densitometry	HSV-1 protein levels in HFF cells treated with Vehicle, UNC0379 or NSC663284					3 experiment; 6 replicate samples	6
**S3**		MTT assay	MTT toxicity assay of HFF cells treated with Vehicle, UNC0379 or saponin.					4 experiment; 12-13 replicate samples	4
**S4**	**A**	NSC663284 titration	HSV-1 mRNA levels in HFF cells treated with Vehicle or NSC663284					3 experiment; 6 replicate samples	6
**S4**	**B**	MTT assay	MTT toxicity assay in HFF cells treated with Vehicle or NSC663284					1 experiment; 4 replicate samples	4
**S5**		UNC0379 impact on HCMV expression	mRNA levels in HCMV infected cells treated with Vehicle or UNC0379					3 experiments	3
**S6**	**B**	Western blot densitometry	H4K20me1 proteins levels in HFF cells treated with Vehicle, 4 μM and 8 μM UNC0379 for 5, 24, and 48 h	Unpaired two-tailed t test	K20me1 levels; Vehicle vs 4 μM UNC0379, 5 h<br>K20me1 levels; Vehicle vs 4 μM UNC0379, 24 h<br>K20me1 levels; Vehicle vs 4 μM UNC0379, 48 h<br>K20me1 levels; Vehicle vs 8 μM UNC0379, 5 h<br>K20me1 levels; Vehicle vs 8 μM UNC0379, 24 h<br>K20me1 levels; Vehicle vs 8 μM UNC0379, 48 h	0.1533 ns<br>0.0036 **<br>0.0008 ***<br>0.2906 ns<br>0.0036 **<br>0.0003 ***		5 experiments	5
**Table S2**		Epigenetic inhibitor screen	ICP27 and control cellular mRNA levels in HFF cells treated with Vehicle or indicated inhibitor					2-4 replicate samples	n = 2, 3, or 4

Supplemental Table 4. Statistics. Relevant statistical tests, number of samples or experiment replicates, and p-values of data described in this study.

SUPPLEMENTAL REFERENCES

1. Rozman B, Nachshon A, Levi Samia R, Lavi M, Schwartz M, Stern-Ginossar N. 2022. Temporal dynamics of HCMV gene expression in lytic and latent infections. Cell Rep 39:110653.
2. Liang Y, Vogel JL, Arbuckle JH, Rai G, Jadhav A, Simeonov A, Maloney DJ, Kristie TM. 2013. Targeting the JMJD2 histone demethylases to epigenetically control herpesvirus infection and reactivation from latency. Sci Transl Med 5:167ra5.
3. Ottosen S, Herrera FJ, Doroghazi JR, Hull A, Mittal S, Lane WS, Triezenberg SJ. 2006. Phosphorylation of the VP16 transcriptional activator protein during herpes simplex virus infection and mutational analysis of putative phosphorylation sites. Virology 345:468–81.
4. Arbuckle JH, Gardina PJ, Gordon DN, Hickman HD, Yewdell JW, Pierson TC, Myers TG, Kristie TM. 2017. Inhibitors of the Histone Methyltransferases EZH2/1 Induce a Potent Antiviral State and Suppress Infection by Diverse Viral Pathogens. mBio 8.
5. Cristea IM, Moorman NJ, Terhune SS, Cuevas CD, O'Keefe ES, Rout MP, Chait BT, Shenk T. 2010. Human cytomegalovirus pUL83 stimulates activity of the viral immediate-early promoter through its interaction with the cellular IFI16 protein. J Virol 84:7803–14.
6. Mitchell DP, Savaryn JP, Moorman NJ, Shenk T, Terhune SS. 2009. Human cytomegalovirus UL28 and UL29 open reading frames encode a spliced mRNA and stimulate accumulation of immediate-early RNAs. J Virol 83:10187–97.
7. Terhune SS, Moorman NJ, Cristea IM, Savaryn JP, Cuevas-Bennett C, Rout MP, Chait BT, Shenk T. 2010. Human cytomegalovirus UL29/28 protein interacts with components of the NuRD complex which promote accumulation of immediate-early RNA. PLoS Pathog 6:e1000965.
8. Alfonso-Dunn R, Arbuckle JH, Vogel JL, Kristie TM. 2020. Inhibition of the Super Elongation Complex Suppresses Herpes Simplex Virus Immediate Early Gene Expression, Lytic Infection, and Reactivation from Latency. mBio 11.
9. Cliffe AR, Knipe DM. 2008. Herpes simplex virus ICP0 promotes both histone removal and acetylation on viral DNA during lytic infection. J Virol 82:12030–8.
10. Hill JM, Quenelle DC, Cardin RD, Vogel JL, Clement C, Bravo FJ, Foster TP, Bosch-Marce M, Raja P, Lee JS, Bernstein DI, Krause PR, Knipe DM, Kristie TM. 2014. Inhibition of LSD1 reduces herpesvirus infection, shedding, and recurrence by promoting epigenetic suppression of viral genomes. Sci Transl Med 6:265ra169.
11. Alfonso-Dunn R, Turner AW, Jean Beltran PM, Arbuckle JH, Budayeva HG, Cristea IM, Kristie TM. 2017. Transcriptional Elongation of HSV Immediate Early Genes by the Super Elongation Complex Drives Lytic Infection and Reactivation from Latency. Cell Host Microbe 21:507–517 e5.
12. Arbuckle JH, Kristie TM. 2014. Epigenetic repression of herpes simplex virus infection by the nucleosome remodeler CHD3. mBio 5:e01027–13.
13. Cohen C, Corpet A, Roubille S, Maroui MA, Poccardi N, Rousseau A, Kleijwegt C, Binda O, Texier P, Sawtell N, Labetoulle M, Lomonte P. 2018. Promyelocytic leukemia (PML) nuclear bodies (NBs) induce latent/quiescent HSV-1 genomes chromatinization through a PML NB/Histone H3.3/H3.3 Chaperone Axis. PLoS Pathog 14:e1007313.
14. Frietze S, O'Geen H, Blahnik KR, Jin VX, Farnham PJ. 2010. ZNF274 recruits the histone methyltransferase SETDB1 to the 3' ends of ZNF genes. PLoS One 5:e15082.
15. Tanaka H, Takebayashi SI, Sakamoto A, Igata T, Nakatsu Y, Saitoh N, Hino S, Nakao M. 2017. The SETD8/PR-Set7 Methyltransferase Functions as a Barrier to Prevent Senescence-Associated Metabolic Remodeling. Cell Rep 18:2148–2161.
16. Kristie TM, Pomerantz JL, Twomey TC, Parent SA, Sharp PA. 1995. The cellular C1 factor of the herpes simplex virus enhancer complex is a family of polypeptides. J Biol Chem 270:4387–94.
